# A DNA structural alphabet provides new insight into DNA flexibility

**DOI:** 10.1107/S2059798318000050

**Published:** 2018-01-01

**Authors:** Bohdan Schneider, Paulína Božíková, Iva Nečasová, Petr Čech, Daniel Svozil, Jiří Černý

**Affiliations:** a Institute of Biotechnology of the Czech Academy of Sciences, BIOCEV, Průmyslová 595, CZ-252 50 Vestec, Czechia; b Laboratory of Informatics and Chemistry, Faculty of Chemical Technology, University of Chemistry and Technology Prague, Technická 5, CZ-166 28 Prague, Czechia

**Keywords:** DNA structure, X-ray structure, NMR structure, DNA modelling, bioinformatics

## Abstract

DNA is a structurally plastic molecule, and its biological function is enabled by adaptation to its binding partners. To identify the DNA structural polymorphisms that are possible in such adaptations, the dinucleotide structures of 60 000 DNA steps from sequentially nonredundant crystal structures were classified and an automated protocol assigning 44 distinct structural (conformational) classes called NtC (for Nucleotide Conformers) was developed. To further facilitate understanding of the DNA structure, the NtC were assembled into the DNA structural alphabet CANA (Conformational Alphabet of Nucleic Acids) and the projection of CANA onto the graphical representation of the molecular structure was proposed. The NtC classification was used to define a validation score called confal, which quantifies the conformity between an analyzed structure and the geometries of NtC. NtC and CANA assignment were applied to analyze the structural properties of typical DNA structures such as Dickerson–Drew dodecamers, guanine quadruplexes and structural models based on fibre diffraction. NtC, CANA and confal assignment, which is accessible at the website https://dnatco.org, allows the quantitative assessment and validation of DNA structures and their subsequent analysis by means of pseudo-sequence alignment. An animated Interactive 3D Complement (I3DC) is available in Proteopedia at http://proteopedia.org/w/Journal:Acta_Cryst_D:2.

## Abstract 2

Large deformations of the DNA double helix induced by interactions with proteins and small molecules are necessary to support the biological function of DNA. Here, the software tools available at https://dnatco.org that classify the dinucleotide building blocks into 44 distinct structural classes and 11 letters of a first DNA structural alphabet are presented and are used to analyze several prototypical DNA structures.

## 1. Introduction   

The prevailing DNA architecture, a right-handed double helix composed of sequentially complementary antiparallel strands, is structurally very plastic. Local deformations of DNA induced by interactions with binding partners, proteins and small-molecule drugs are necessary to convey its biological function, conservation and transfer of genetic information. The DNA strand has the ability to accommodate large conformational changes by forming kinks or bends or to undergo radical rearrangements into loops or folded forms such as quadruplexes. These conformational changes cannot be understood without going beyond the ‘A–B–Z’ classification traditionally used to describe DNA structural diversity. The necessity of understanding the DNA conformational space in its full complexity increases with the increasing number of biologically important DNA structures other than the double helix: tetraplexes with complicated and variable topologies, single-stranded hairpins and cruciforms, and DNA junctions involved in recombinant processes such as Holliday junctions.

A detailed analytical view of the backbone conformational behaviour is not easy to obtain. The analysis is complicated by two factors: the plasticity of the DNA molecule, which leads to a host of DNA conformers, and the many torsional degrees of freedom of the DNA backbone. The flat hypersurface of potential energy and its multidimensionality make mathematically rigorous analysis of the DNA conformational space hard to accomplish, and a brute-force computational classification of DNA conformers has proven to be futile. The existence of several thousand experimental DNA structures solved by crystallographers and NMR spectroscopists has made such a comprehensive knowledge-based analysis of DNA conformational space possible.

Here, we present a substantial revision of our previously published classification protocol of dinucleotide conformers (Svozil *et al.*, 2008 ▸), leading to the discovery of new conformers occurring in several nonduplex structures such as tetraplexes, four-way junctions and various single-stranded topologies. Analysis of the currently available crystal structures resulted in the establishment of an ensemble of 44 dinucleotide conformers NtC (**N**ucleo**t**ide **C**onformers). The NtC classes were further used to formulate the DNA structural alphabet CANA (**C**onformational **A**lphabet of **N**ucleic **A**cids), a concept that is well known in protein structural analysis (Unger *et al.*, 1989 ▸; Levitt, 1992 ▸), but, to the best of our knowledge, is the first formally defined DNA structural alphabet. Its usefulness has already been demonstrated by analyzing the structural features of DNA complexed with transcription factors and histone proteins (Schneider *et al.*, 2017 ▸). We briefly characterize the NtC conformers and show their typical occurrence in DNA structures, and use the NtC classification to calculate a validation score called confal, which allows quantification of the agreement between the known conformers and an analyzed dinucleotide. We then demonstrate the usefulness of the NtC and CANA classification scheme by annotating a few prototypical DNA structures.

## 2. Methods   

### 2.1. Retrieval of sequentially nonredundant structures   

We retrieved two sets of crystal structures: 2405 protein–DNA complexes and 879 structures of ‘naked’ DNA. To capture as wide a spectrum of DNA structures as possible, we selected structures with a crystallographic resolution equal to or better than 3.0 Å by a search in the Protein Data Bank (PDB; Berman, Battistuz *et al.*, 2002 ▸) and the Nucleic Acid Database (Berman, Westbrook *et al.*, 2002 ▸) (release of 9 April 2014). We rejected structures containing RNA or DNA/RNA hybrids. Further, we excluded structures that did not contain DNA longer than five nucleotides or peptides longer than 20 amino acids. This criterion led to the exclusion of 173 protein–DNA complexes and 149 structures of naked DNA. To avoid the analysis of sequentially redundant structural data, we excluded structures with similar protein and at the same time similar DNA sequences, keeping just one structure with the highest crystallographic resolution in the case where it was better by 0.2 Å or more or otherwise that with the best *MolProbity* score (Chen *et al.*, 2010 ▸). The treatment of sequence redundancies is described in more detail in the Supporting Information. The final data sets contained 58 069 steps of natural dinucleotides obtained from 1389 protein–DNA complexes and 413 structures of naked DNA.

### 2.2. The conformational assignment of steps   

**Figure 1:**
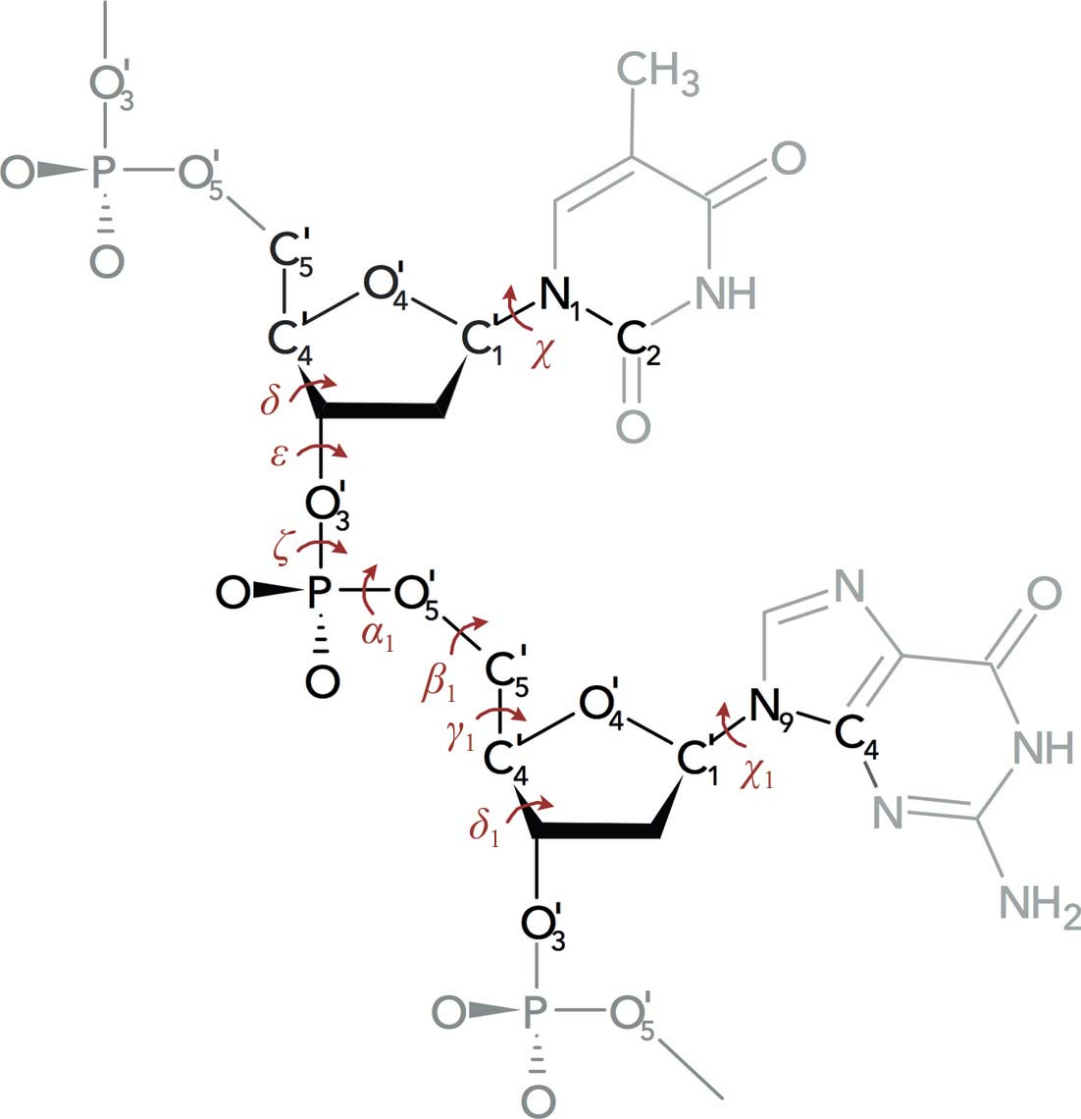
The analyzed DNA fragment, a dinucleotide without its 5′ phosphate, is drawn in black and is referred to as a step. Its structure is represented by the nine torsion angles δ, ∊, ζ, α_1_, β_1_, γ_1_, δ_1_, χ and χ_1_; the atoms defining the torsions are labelled. dTG is shown here; all 16 possible dinucleotide sequences were included in the study.

We analyzed structures of a DNA fragment between C5′ of one nucleotide and O3′ of the following nucleotide containing two deoxyriboses, two bases and the linking phosphate group. This is depicted in Fig. 1 ▸ and is referred to as a step. The step conformations were analyzed in nine-dimensional torsion space defined by seven backbone torsions labelled δ, ∊, ζ, α_1_, β_1_, γ_1_ and δ_1_, and two torsions around the glycosidic bond, χ and χ_1_. The stable conformations referred to as NtC (Nucleotide Conformers) were similarly to those in our previous study (Čech *et al.*, 2013 ▸) assigned by the method of weighted *k* nearest neighbours (*k*-NN). This approach requires the availability of a training data set during the prediction phase. As the training set, we initially used step data consisting of manually checked assignments into the 18 NtC used in the original *k*-NN protocol.

### 2.3. Enhancement of the original *k*-NN protocol and the discovery of new NtC classes   

The application of the original *k*-NN protocol (Čech *et al.*, 2013 ▸) to the current data led to the assignment of 50 136 steps; 7903 remained unassigned. These were further analyzed by complete linkage hierarchical clustering with a circular Euclidean distance implemented in the *R* statistical software. This analysis reassigned several thousand steps of the original 18 NtC and discovered 26 new NtC classes. In total, there were 8472 reassigned and newly assigned steps. The identification of new NtC led to a new training set, referred to in the following as the golden set. The resulting golden set contains a manually curated self-consistent ensemble of 4431 steps that define the 44 currently specified NtC classes (Table 1 ▸; the full list is given in Supplementary Table S1). The majority of the steps in the golden set originated from structures with resolution better than 2.0 Å, but some conformers needed to be defined, at least in part, by structures with worse resolutions (Supplementary Fig. S2).

There have been a few changes in the details of the description of the conformational classes since the web server https://dnatco.org was set up (Černý *et al.*, 2016 ▸): the conformational classes NtC were originally called ntC and their number diminished from 57 to 44 after merging formally distinguishable but nevertheless structurally close clusters. The website https://dnatco.org contains both the original and the current version of the assignment.

### 2.4. The final assignment protocol   

The final assignment protocol, which is described in greater detail in the Supporting Information and Supplementary Fig. S1, can be summarized as follows.

(i) An uploaded PDB-formatted file is analyzed for the presence of DNA steps, and their torsions are calculated.
(ii) The distances between torsions of a candidate step and all members of the golden set are calculated and sorted. The distances are measured as the sum of circularly measured Euclidean distances *d* between torsions of the candidate and the golden-set members. In our weighted *k*-NN protocol, the distance weights are calculated as 1/*d* ^2^ so that nearest neighbours contribute more than distant ones.
(iii) A conformation of the candidate is assigned to the NtC class of the nearest neighbour from a list of up to 11 but no less than seven nearest neighbours. If the sum of the weighted distances between the candidate and its nearest neighbours belonging to the same NtC class is less than 0.0011, the candidate is declared as unclassified and is formally assigned to the NAN class. The limit of 0.0011 (1/30^2^) is an empirically determined limit that offers a reasonable ratio between the number of assigned and unassigned steps.
(iv) In addition, a candidate is not classified if it fails any of the following tests.
  (1) Each of the nine torsions of a candidate must lie within an empirically determined distance of 28° from the actual values of the corresponding torsions of the nearest neighbour and from the averages of the corresponding torsions of its nearest neighbours from the golden set belonging to the same NtC class.
  (2) Each torsion of a candidate must lie within the empirically determined value of five estimated standard deviations from the averages of the corresponding torsions of the NtC class from the golden set.
  (3) To minimize the influence of a systematic difference between the torsion values of a candidate and the torsion averages of NtC in the golden set, the sum of the signed differences must not differ by more than ±60° for the seven backbone torsions (δ, ∊, ζ, α_1_, β_1_, γ_1_, δ_1_) and by no more than ±60° for the two glycosidic torsions (χ and χ_1_). The limiting differences of ±60° were selected based on the analysis of sums of differences from the data assigned without applying this test (Supplementary Fig. S3).
  (4) To exclude a possible ambiguous relationship between the torsion angles δ and δ_1_ and the sugar puckers of the analyzed DNA fragment (step), the values of the pseudo­rotation angle *P* (Altona & Sundaralingam, 1972 ▸) of the candidate deoxy­riboses must not differ more than ±72° from the average value of the pseudorotation *P* for the golden set of the tested NtC class.

### 2.5. NtC classes as a tool to validate DNA structures: confal score   

**Figure 2:**
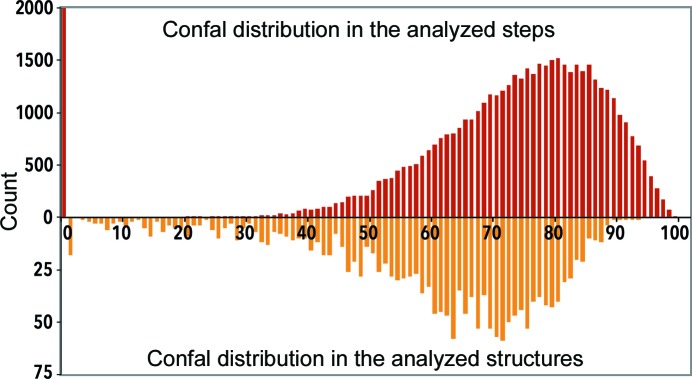
Distributions of the values of the validation score confal. Confal runs from 0 to 100 and is displayed on the horizontal axis: the upper histogram in red plots the confal distribution in all ∼60 000 analyzed steps. The bar at a confal value of 0 indicates 12 557 unassigned steps. The bottom histogram in orange displays the confal distribution of the 1802 analyzed structures.

To quantify the closeness of fit between a candidate step and the assigned NtC class, we introduced a conformational validation score: confal. It is defined as the Gaussian-weighted distance between the torsion values of the candidate and the average for the NtC,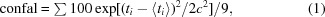 where *t*_*i*_ are the torsion values of the candidate and 〈*t*_*i*_〉 are the corresponding averages of the assigned NtC; the summation runs over the nine step torsion angles. The coefficient *c* ^2^ is calculated for each NtC class in such a way that confal equals 100 for a perfectly matching candidate and 1 at the limiting values of ±28° or ±5 estimated standard deviations from the NtC torsion averages, whichever value is smaller; confal is set to 0 for unassigned candidates. The above formula calculates confal values for steps; the value for the whole structure is the arithmetic average of the confal values of all steps. Fig. 2 ▸ shows the confal distributions for all ∼60 000 steps and in 1802 analyzed structures. Based on the data in Fig. 2 ▸, a percentile rank is reported for any structure analyzed using the web server at https://dnatco.org.

## 3. Results and discussion   

### 3.1. Overview   

**Figure 3:**
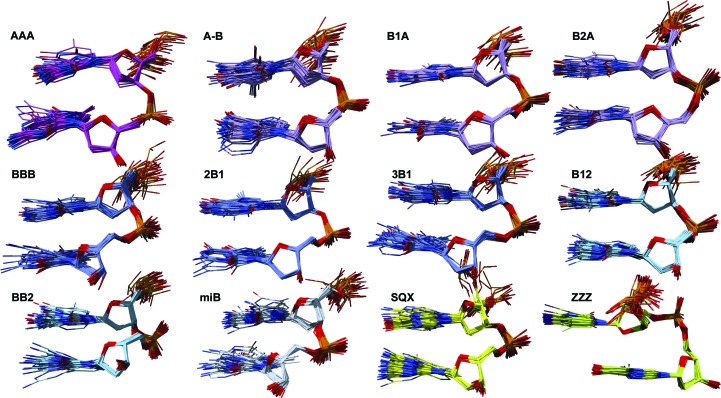
Gallery of the CANA letters. Each letter is represented by 40 randomly selected structures of steps which represent one NtC class belonging to the letter in the golden set. A-like and mixed B/A conformers are drawn in pink and violet, B-like conformers in blue and conformers with either base in the *syn* orientation in green. The CANA letter AAA is represented by NtC AA00, A-B by AB01, B1A by BA01, B2A by BA13, BBB by BB00, 2B1 by BB01, 3B1 by BB02, B12 by BB04, BB2 by BB07, miB by BB10, SQX by BBS1 and ZZZ by ZZ2S. The B1A and B2A letters are merged in Table 1 ▸ to B-A.

We first characterize the structural properties of 44 distinct step-conformer classes called NtC (Nucleotide Conformers; the full list is given in Supplementary Table S1) and introduce a coarser yet comprehensive classification of DNA conformations: a structural alphabet called CANA (Conformational Alphabet of Nucleic Acids; Table 1 ▸, Fig. 3 ▸). In the last section, we discuss the occurrence of NtC and CANA in a few archetypal DNA structures (Fig. 6) and show a potential use of these conformational classifications for the straightforward and objective annotation of DNA structures. An automated assignment of NtC, CANA and confal for any PDB-formatted DNA structure can be carried out at the website https://dnatco.org (Černý *et al.*, 2016 ▸).

### 3.2. Dinucleotide conformers NtC and the DNA structural alphabet CANA   

Our analysis of DNA step structures separated them into 44 distinct conformer classes called NtC that are described in detail in the Supporting Information and Supplementary Table S1. The NtC assignment was implemented in the automated procedure available at https://dnatco.org (Černý *et al.*, 2016 ▸). A fine-grained clustering into 44 classes is convenient for further computational use, but it is perhaps too detailed for straightforward human interpretation. We therefore assembled NtC classes according to their major structural features into 11 groups that define the DNA structural alphabet CANA. A brief annotation of all of the letters of the CANA alphabet in Table 1 ▸ characterizes their main structural features; the twelfth letter is formally ascribed to structurally unassigned conformers. In the following text, we briefly characterize the CANA letters and their constituting NtC classes; more examples with a more detailed description and detailed NtC assignment can be found in the Supporting Information.

#### 3.2.1. AAA: A-form   

The main conformational feature of the A-form is the sugar pucker in the C3′-*endo* region; this is the most frequent A-DNA conformer and is described here as NtC AA00, which is also characterized by a low anti-glycosidic torsion χ of near 200°. It is structurally identical to A-RNA. DNA duplexes classified as A-DNA are conformationally homogeneous; for example the structure with PDB entry 1d91 (Kneale *et al.*, 1985 ▸) is composed almost exclusively of the ‘canonical’ A-DNA NtC AA00.

#### 3.2.2. A-B: conformers bridging A-form to B-form   

**Figure 4:**
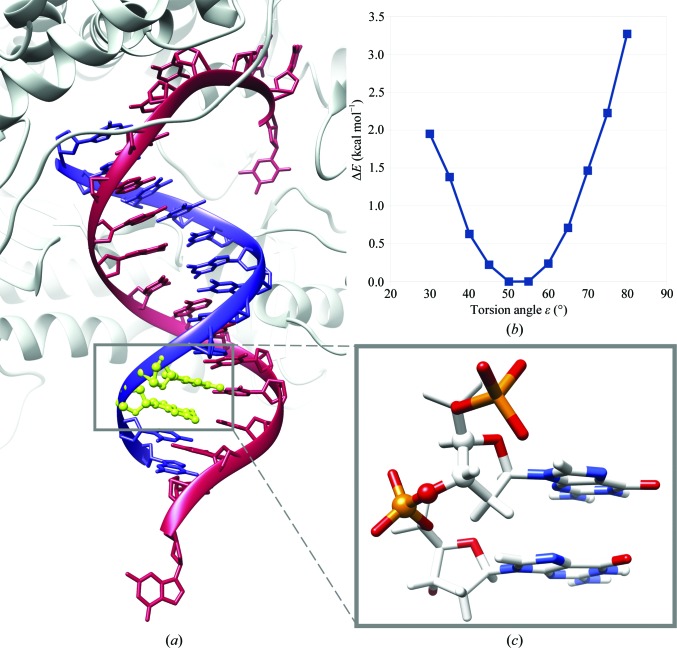
The NtC conformer AB02 is locally stable despite its extremely low ∊ value of ∼60°. (*a*) The AB02 step DG105–DG106 of chain *P* from the crystal structure with PDB code 4fj7 (Xia *et al.*, 2013 ▸) is highlighted in green in cartoon representation and is enlarged in the inset on the right. (*b*) A scan of the potential energy of the step modelled as an isolated dinucleotide with charge −2 is shown on the top right; the ∊ torsion was scanned between 30 and 80°. Quantum-mechanical calculation of the stability of AB02 is detailed in the Supporting Information.

The main A-B conformer, AB01, has been described previously (Svozil *et al.*, 2008 ▸). A newly identified conformer, AB02, is unique owing to its extremely low value of the torsion ∊ of near 60°. Despite the exotic and unexpected value of ∊, AB02 is observed relatively frequently in more than 100 structures. To test the stability of AB02, we performed quantum-mechanical calculations, which are summarized in Fig. 4 ▸ and described in the Supporting Information and Supplementary Table S2. The calculations showed that a dinucleotide with value of the torsion ∊ of near 60° is at its local minimum, making the conformer an unlikely artifact of intermolecular interactions. The ∊ value at the energy minimum coincides with the ∊ values observed in the crystal and that calculated for the AB02 class average (Supplementary Table S2).

#### 3.2.3. B-A: conformers bridging B-form to A-form   

B-to-A conformers represent a structural complement of the previous CANA letter, A-B, but they are represented by a much more diverse and numerous group of conformers than A-B. All B-to-A conformers occur in duplexes, but they can also occur in quadruplexes (BA13 in PDB entry 3sc8; Collie *et al.*, 2012 ▸) or joints of Holliday junctions (BA05 in PDB entry 1p4e; Chen & Rice, 2003 ▸). Most of them (NtC BA01–BA10) can be characterized as BI-to-A, while conformers that describe a transition between BII and A forms (BA13–BA17) are much rarer.

#### 3.2.4. BBB, 2B1, 3B1, B12, BB2 and miB: conformers that can be characterized as B-like   

What is usually described as the ‘B-DNA form’ should be seen as a family of conformations rather than a single, firmly defined structure. The two traditionally recognized B forms, BI and BII, also do not suffice to describe the conformational diversity of the B-like backbone. In the structural alphabet CANA, we describe the B-form by three letters for BI conformers (BBB, 2B1 and 3B1), one letter for BII conformers (BB2) and one letter for transitional BI-to-BII conformers (B12). In addition, we grouped numerically minor but structurally diverse conformers that still bear some features of the B-form under the letter miB (for mixed B-DNA).

#### 3.2.5. BBB, 2B1 and 3B1: letters describing the BI form   

The dominant DNA conformer is NtC BB00, representing about one third of all analyzed steps. Therefore, it alone constitutes CANA letter BBB. It should be viewed as the canonical BI-form. Dinucleotides whose conformations belong to the letters BBB, 2B1 and 3B1 form the core of Watson–Crick-paired double helices, but they can occur in almost any structural context, including quadruplexes or joints of Holliday junctions. This adaptability to various local constraints further demonstrates the role of BI as the main B-DNA conformation.

#### 3.2.6. BB2 and B12: BII and BI-to-BII   

The main characteristics that distinguish BI and BII forms is a switch of the values of the ∊ and ζ torsions from 180 and 260° in BI to about 250 and 180° in BII, respectively. The most frequent conformer epitomizing the BII form is BB07. CANA letter B12 represents conformers with torsion-angle values between those typical of the BI and BII forms. Both BB2 and B12 occur especially at positions where the DNA duplex accommodates its shape to the interacting protein.

#### 3.2.7. miB: various B-like conformers   

The high plasticity of B-DNA is demonstrated by the existence of several distinct conformers that, despite their variability, share the typical features of the B-form: both sugars in the C2′-*endo* pucker and both glycosidic torsions χ above 200°. The structural features of these conformers can be quite exotic, but they have often been modelled with base stacking in double helices. If modelled correctly, they are likely to be energetically un­favourable but perhaps possible if stabilized by other strong interactions. The most important member of the miB letter is the NtC class BB16. It is important owing to its ability to accommodate a large local deformation of the strand. The particular combination of torsions attributable to this conformer can lead to unstacking of the bases to incorporate an intercalated drug (PSB entry 1p20; Howerton *et al.*, 2003 ▸); BB16 is also often found at or adjacent to the strand crossing in Holliday junctions (PDB entry 1l4j; Thorpe *et al.*, 2003 ▸). BB14 is similar to AB02, which was discussed above, in its extremely low value of the ∊ torsion.

#### 3.2.8. SQX: conformers occurring mainly in nonduplex DNA   

Conformers grouped into this structurally variable CANA letter are rare but important for the architecture of folded forms of DNA, because steps in these conformations are found in unpaired or mismatched parts of DNA duplexes or in nonduplex DNA structures. NtC AB1S, BBS1, BB2S and NS1S occur in guanine tetraplexes, while BB1S and BB2S are observed only in their G–G steps. BBS1 not only forms the G-quartet core of quadruplexes (PDB entry 1jpq; Haider *et al.*, 2002 ▸), but it also accommodates a purine–purine mismatch (PDB entry 178d; McAuley-Hecht *et al.*, 1994 ▸) and occurs in the loops of complicated self-folding single-stranded hairpin structures (PDB entry 2vju; Barabas *et al.*, 2008 ▸).

#### 3.2.9. ZZZ: conformers describing the Z-form   

The left-handed Z-DNA duplex is built from steps belonging to four NtC classes. ZZS1 and ZZS2 describe the purine–pyrimidine steps; the alternating pyrimidine–purine steps are described by ZZ1S and ZZ2S. ZZS1 and ZZ1S are more frequent, forming the so-called ZI-DNA. Two minor NtC classes, ZZ2S and ZZS2, which have δ_1_ in the C2′-*endo* pucker and can only occur at strand ends, form the ZII-DNA form.

#### 3.2.10. NAN: non-assigned conformers   

About 21% of the analyzed steps end up as unassigned to any NtC with defined torsion values and are labelled NAN. Although we aimed at the highest possible percentage of assigned steps, we considered a fraction of 21% of unclassified steps to be a realistic compromise between the accuracy of assignment and the conformational properties of the analyzed steps. Therefore, we decided not to loosen up the parameters of the assignment process to classify more steps.

We find two reasons why steps remain structurally uncharacterized: (i) a missing template in the golden set and (ii) incorrect model building during the refinement process. While the latter reason can be remedied by the use of improved force fields for model building and by more robust validation protocols, the former is caused by as yet incomplete sampling of the nine-dimensional conformational space of the DNA backbone. To estimate the contributions of the two reasons is not easy, but some facts point to a large proportion of incorrectly refined structures. A significant majority of unassigned steps (over 90%) were found in right-handed double-helical structures with Watson–Crick paired bases, where we do not see any reason for the massive occurrence of so far unknown conformers. We assume that most of these steps are likely to be a consequence of insufficiently refined duplexes, as can, for example, be seen in the dodecamers of PDB entries 1vte (Aymami *et al.*, 1990 ▸) and 1dne (Ala-Kokko *et al.*, 1989 ▸), which have no or very few of their steps structurally assigned (Supplementary Table S3). Furthermore, comparison of the conformers reported in this and our previous analyses (Svozil *et al.*, 2008 ▸; Čech *et al.*, 2013 ▸) suggests that the majority have already been characterized, and that newly discovered conformer classes will be numerically small, accounting for a small fraction of the currently unassigned steps.

The issue of incorrectly refined structures has been approached by Sunami *et al.* (2017 ▸), who found that about a quarter of phosphate groups in higher resolution structures are actually disordered and may be positioned better in the electron density. The authors of the *PDB_REDO* web service (Joosten *et al.*, 2009 ▸) offer an automated procedure to optimize crystallographic structure models deposited in the PDB. To see how *PDB_REDO* changes the distributions of the assigned NtC classes, we compared the NtC distributions in DNA structures as they had been deposited in the PDB and after they had been treated by *PDB_REDO*. As a test case, we selected 34 structures of dodecamers related to the Dickerson–Drew dodecamer. On average, the confal score improved by 7 after application of the *PDB_REDO* protocol, but the fraction of reassigned NtC classes varied widely between 0 and 77% in individual structures. The confal values showed significant improvement, for example, in the structure with PDB entry 1d29 (Larsen *et al.*, 1991 ▸), while no changes were observed in PDB entry 4i9v (Szulik *et al.*, 2015 ▸) and a mixture of increased and lowered confal values were found in PDB entry 5bna (Wing *et al.*, 1984 ▸) (Supplementary Table S3). Overall, the *PDB_REDO* protocol improves the deposited coordinates somewhat, but better molecular templates provided by the NtC classes can constrain the re-refinement more effectively.

#### 3.2.11. How well is the sugar pucker described by the backbone torsion δ?   

**Figure 5:**
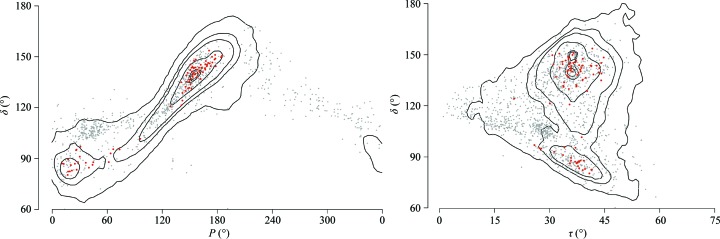
Scattergrams relating the backbone torsion δ and the deoxyribose pucker described by the pseudorotation phase angle *P* and magnitude τ. The contours enclose 99.9, 90, 50, 20, 10 and 5% of the assigned steps. Red dots represent the average δ, *P* and τ values of the NtC classes as they are defined in the golden set. Grey diamonds show the values for unassigned dinucleotides. Only a few deoxyriboses have δ within the physically possible limits 75–165° and *P* outside the −30 to 210° region associated with C3′-*endo* and C2′-*endo* sugar puckers. As expected, the magnitude τ does not discriminate between different sugar puckers. In both graphs, many unassigned dinucleotides acquire atypical δ–*P* and δ–τ combinations and the respective deoxyriboses are in all likelihood not modelled correctly.

The sugar pucker is correctly described by two parameters, the pseudorotation phase angle *P* and its magnitude τ (Altona & Sundaralingam, 1972 ▸), but we simplify its description and use just one parameter: the torsion angle δ. A potential problem of this simplification is represented by the ambiguous relationship between *P* and δ, where one value of δ may correspond to two *P* values describing two different sugar puckers. As Fig. 5 ▸ demonstrates, situations in which δ indicates the C2′-*endo* or C3′-*endo* pucker but *P* proves otherwise are rare even for unassigned steps. Most importantly, the scattergrams in Fig. 5 ▸ show that the δ torsion represents the sugar pucker correctly in the golden set: the region around the C3′-*endo* pucker with *P* values of 0–60° is indeed associated with δ ≃ 80° and the region around the C2′-*endo* pucker with *P* = 150–180° is associated with δ ≃ 130°. The condition that *P* of a classified step must fall within ±72° of the golden-set *P* average (see §[Sec sec2]2) led to the exclusion of ∼0.8% (484) of the analyzed steps in the whole set. Of interest might be the golden-set averages near *P* = 60° corresponding to the O4′-*endo* sugar pucker. As expected, the magnitude τ varies in a narrow range and does not discriminate between different sugar puckers.

### 3.3. NtC classes as a tool to validate DNA conformations: confal score   

The confal score of a step measures how closely its structure matches the structure of the NtC class to which it was assigned. The confal distribution for the analyzed steps (upper histogram in Fig. 2 ▸) shows two features: (i) a smooth distribution for the assigned steps with a mode value of about 80 and few steps with confal values below 30, and (ii) a tall peak for ∼12 000 unassigned steps with confal 0. In contrast, the confal distribution of individual structures is shifted to lower values. While most structures have confal values of between 60 and 80, and a structure with a confal value of about 65 is in the 50th percentile (median), the distribution has a long tail that extends to values below 10. This indicates a very poor match between the geometries of the NtC classes and steps in some of the analyzed structures. Because confal quantifies the conformity between the geometries of analyzed steps and of the defined NtC classes, a particular value of confal and the corresponding percentile do not necessarily mean high or low structure quality; it just shows the similarity to the known conformers. For instance, the Dickerson–Drew dodecamer of PDB entry 1bna (Drew *et al.*, 1981 ▸) has confal 77 and belongs to the 84th percentile among the classified structures, indicating that the steps of this structure can be well characterized by the known conformers.

### 3.4. Annotation of a few selected prototypical DNA structures   

**Figure 6:**
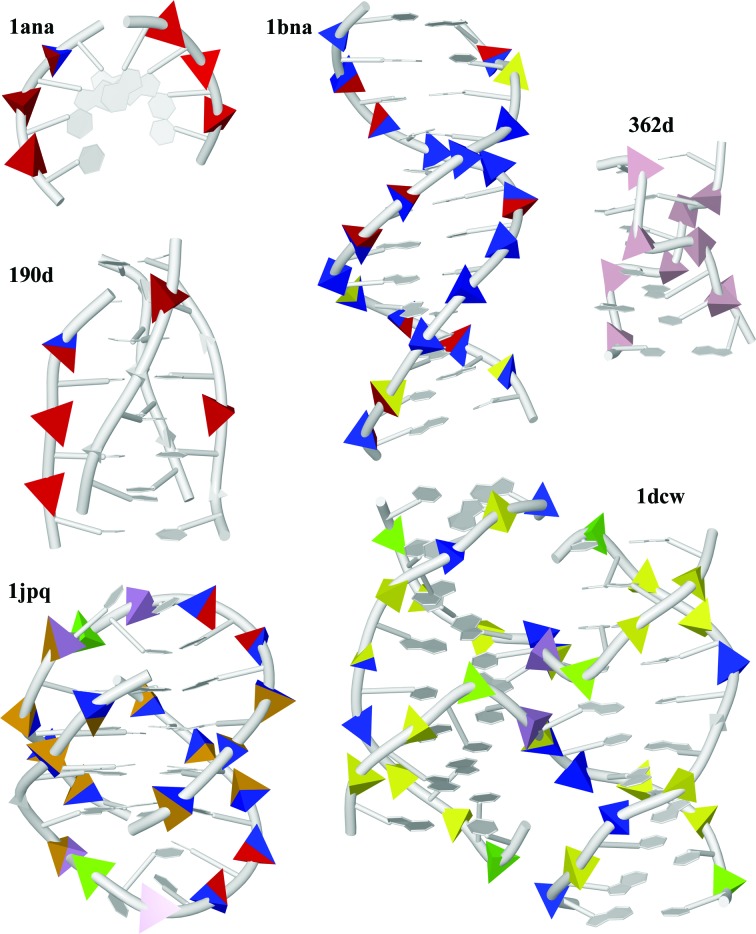
Graphical representation of the CANA letters assigned to DNA structures. Each phosphate group is enclosed with a colour-coded tetrahedron representing the assigned CANA letter: letter AAA is labelled red, BBB, 2B1 and 3B1 blue, BB2 yellow, miB green, ZZZ pink, steps with bases in the *syn* orientation orange and unassigned steps (NAN) grey. Steps that mix the features of two letters are composed of two colours: for example, a step mixing BII and A forms is shown in yellow/red. The sizes of the tetrahedrons represent the confal values for each step. The tetranucleotide with PDB entry 1ana (Conner *et al.*, 1984 ▸) is a typical A-DNA, PDB entry 1bna (Drew *et al.*, 1981 ▸) is B-DNA overall but with pronounced A-like features, PDB entry 362d (Harper *et al.*, 1998 ▸) is a typical Z-­DNA, and PDB entry 190d (Chen *et al.*, 1994 ▸) is DNA folded as an i-motif with several A-like steps but with most steps unassigned. The quadruplex with PDB entry 1jpq (Haider *et al.*, 2002 ▸) has most steps in the B-DNA form; some steps have *syn* bases assigned (in orange) and a few are not assigned. The Holliday junction with PDB entry 1dcw (Eichman *et al.*, 2000 ▸) is formed by four stems with a prevailing BII-DNA form; two steps at the junction have unstacked bases and are assigned the letter SQX (in violet), while just one step is not assigned. A tabular form of the NtC and CANA assignment of the steps in these six structures can be found in Supplementary Table S3; any DNA structure can be visualized at https://dnatco.org.

Fig. 6 ▸ displays a graphical representation of the CANA letters assigned to a few DNA structures. The representation provides a concise way to visualize the main structural features of a DNA structure. For instance, alterations of B-like and A-like features are visible in PDB entry 1bna and the presence of two steps with unstacked bases at the junction of a four-way junction is visible in PDB entry 1dcw (Eichman *et al.*, 2000 ▸). A few structure types are discussed in more detail below. A more detailed discussion of the structural features of DNA bound to two specific classes of proteins, transcription factors and histone proteins, can be found in a recently published study (Schneider *et al.*, 2017 ▸).

#### 3.4.1. The Dickerson–Drew dodecamer   

The DNA do­decamer with the palindromic sequence d(CGCGAATTCGCG), often called the Dickerson–Drew dodecamer (D-Dd), was the first solved crystal structure of a right-handed DNA duplex (Drew *et al.*, 1981 ▸) and has often been assumed to represent a prototypical B-DNA duplex. The importance of D-Dd is documented by the fact that a host of related structures were subsequently solved with gradually increasing resolution, it has been co-crystallized with various drugs, its components have been chemically modified and the structures of many oligonucleotides possess sequences related to D-Dd. D-Dd has also served as a test bed for *in silico* modelling since the first force-field simulations (Kollman *et al.*, 1982 ▸). We therefore believe that a conformational analysis of D-Dd-related structures may be of general interest.

We assigned NtC to structures sequentially related to D-Dd solved by both crystallography and NMR; the results are listed in Supplementary Table S3(*b*). A notable aspect of the assignment to the crystal structures is that actually only about half of the steps adopt BI conformations; a full 20% correspond to the CANA letter B-A, 15% are BII or mixed BI/BII (BB2 and B12, respectively) and finally 10% are A and A-B forms. The original D-Dd structure (PDB entry 1bna; Drew *et al.*, 1981 ▸) has nine steps in BI, three in BII, eight in B-to-A and two in A-to-B conformations. An in-depth analysis of sequence-related structural features would require further experimental data on related sequences besides the closely related D-Dd sequences or systematic crystal data on CC*XXX*­NNNGG decamer sequences (Hays *et al.*, 2005 ▸). Just a quick comparison of the central AATT tetranucleotide from the D-Dd structures showed an encouraging convergence of the assigned NtC classes. The AA and TT steps converge to BB01 and the central AT step to the BA05 NtC class in structures with increasing crystallo­graphic resolution.

Complexation of D-Dd-related oligonucleotides with minor groove-binding drugs does not induce significant conformational changes; these structures show similar statistics just with fewer B-A and A-B letters and more non-assigned conformers. The statistics listed in this paragraph do not include a few structures whose steps were impossible to classify because of severe backbone deformation that was likely to have resulted from incomplete refinement.

A different distribution of NtC classes can be seen for D-Dd structures determined in solution by NMR techniques. These structures report many more BI conformers than the crystal structures, but surprisingly no structures described by the A-B, BB2 and B12 letters. The question remains whether these statistics reflect the actual conformational preferences of the D-Dd sequence in solution or are a consequence of the force fields used in NMR refinement.

To quantify the distribution of the backbone conformers in computer simulations of D-Dd, we analyzed the trajectories of several MD simulations (Supplementary Table S3) based on three force fields. Despite detailed discussion of this topic being outside the scope of this study, we conclude that the force fields optimized for long MD simulations of DNA do reproduce the main D-Dd structural features quite realistically. The closest match between the distribution of NtC in crystal structures and MD simulations is observed in short 100 ns simulations (Černý *et al.*, 2008 ▸) and long 1 ms simulations (Galindo-Murillo *et al.*, 2016 ▸) with the bsc0 force field (Pérez *et al.*, 2007 ▸); the bsc1 force field (Ivani *et al.*, 2016 ▸) and OL15 (Zgarbová *et al.*, 2015 ▸) force field underestimate the frequency of B/A forms (AB01, BA06) and overemphasize that of BII (BB07).

#### 3.4.2. Intercalating and *cis*-platinum drugs   

Intercalation of a drug molecule between two base pairs significantly increases their mutual distance. The corresponding deformation of the backbone may or may not be captured as a particular conformer; in most cases, steps with the intercalated drug are unassigned. This is the case for PDB entry 1da0, the hexa­nucleotide CGATCG intercalated with daunomycin (Moore *et al.*, 1989 ▸), but the same sequence with adriamycin, PDB entry 1d12 (Frederick *et al.*, 1990 ▸), fits fully into a combination of B conformers (2×BB2, 2B1 and BBB). Another daunomycin-intercalated hexamer, PDB entry 308d (Gao *et al.*, 1997 ▸), has an unassigned step on one side of its two intercalations, but the other strand uses an unambiguously well fitted BB16, an NtC which also supports glycerol intercalation for the step 312–313 of PDB entry 1mow chain *B* (Chevalier *et al.*, 2002 ▸). *Cis*-platinum and its derivatives are important DNA-binding anticancer drugs. The structures with PDB codes 1ihh (Spingler *et al.*, 2001 ▸) and 1lu5 (Silverman *et al.*, 2002 ▸) report dodecamers with *cis*-platinum-related compounds. The bonds between Pt^2+^ and two guanine bases from the same strand induce a double strand with most steps in the A-form and some unclassified; only a few remain in B conformations.

#### 3.4.3. Guanine tetraplexes   

Guanine tetraplexes have emerged as important noncanonical DNA structures in genomes. G-tetraplex structures are present inside cells (Han & Hurley, 2000 ▸), and bioinformatic evidence has provided insight into the sequence diversity in intronic regions of the human genome (Todd & Neidle, 2011 ▸). The basic building block of G-quadruplexes is a guanine tetrad, in which four guanine bases form a square-planar structure stabilized by the presence of potassium or sodium cations. G-quadruplexes exhibit various topologies characterized as intramolecular, bimolecular and tetramolecular, and are typified by the presence of a large number of various conformers. The proportion of the canonical BI conformation can be as high as 50%, as in the structure of the tetramolecular quadruplex with PDB entry 1o0k (Clark *et al.*, 2003 ▸), where BI steps are interspersed by a few B-A conformers. Steps with guanines in the *syn* orientation acquire NtC such as BBS1, BB1S and BB2S. Dinucleotides within the loops in bimolecular (PDB entry 1jpq; Haider *et al.*, 2002 ▸) and intramolecular (PDB entry 1kf1; Parkinson *et al.*, 2002 ▸) quadruplexes are structurally more flexible and commonly unstacked, and only some of them can currently be classified.

#### 3.4.4. I-motifs   

I-motifs are DNA quadruplexes formed in cytosine-rich sequences (Gehring *et al.*, 1993 ▸) that are comprised of two parallel-stranded duplexes held together in an antiparallel orientation by intercalated hemiprotonated cytosine–cytosine base pairs. In terms of backbone conformations, i-motifs are a rich source of steps in unusual conformations. In cases when their strands consist only of cytosine (PDB entry 190d; Chen *et al.*, 1994 ▸), less than half of the steps adopt AA02, an A-like conformer with BI-like χ/χ_1_, but the rest cannot be assigned to any existing conformational class. They will possibly be classified when more i-motif structures have been solved.

#### 3.4.5. Holliday junctions   

A Holliday junction is a DNA intermediate in homologous recombination (Liu & West, 2004 ▸). It contains four double-stranded DNA arms joined together by short links. The stems of the junctions are usually B-DNA duplexes with very few, if any, B-A and A-B conformers (PDB entry 1dcw; Eichman *et al.*, 2000 ▸). The NtC class that can be associated with the junction proper is the recurrently occurring NS04, but they may be formed by steps in BII BB07, atypical B-like BB16 or unclassified conformations.

### 3.5. DNA models derived from fibre diffraction   

**Figure 7:**
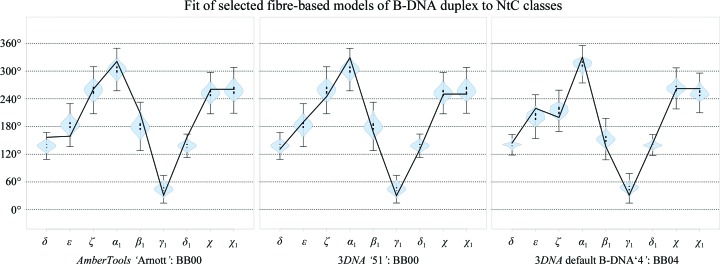
NtC classes assigned to three B-DNA duplex models derived from fibre data: the ‘Arnott’ model from *Nucleic Acid Builder* of *AmberTools*14 (Salomon-Ferrer *et al.*, 2013 ▸) and the models numbered ‘51’ and ‘4’ from 3*DNA* (Lu & Olson, 2008 ▸). The black line connects torsion values for the particular model. The small blue histograms (‘violin plots’) show the distributions of the torsion values of the assigned NtC in the golden set; the vertical grey bars show their total span. A detailed explanation of the graphs is given in Černý *et al.* (2016 ▸) and at https://dnatco.org. The assignment of more fibre models is shown in Supplementary Fig. S4 and Table S5.

DNA models based on the fibre diffraction of DNA are often used as starting structures in computer modelling and other studies. We therefore decided to analyze the backbone conformations of the fibre models available in the 3*DNA* software package (Lu & Olson, 2008 ▸) and in *Nucleic Acid Builder*, which is part of the *AmberTools*14 software package (Salomon-Ferrer *et al.*, 2013 ▸). The assignments of the ‘Arnott’ model from *AmberTools* and of models ‘51’ and ‘4’ from 3*DNA* are shown in Fig. 7 ▸; Supplementary Fig. S4 shows more examples, and the assignment is summarized in Supplementary Table S5. Model 4, which is treated in 3*DNA* as the default B-DNA duplex, is the medium-populated BB04 class with mixed BI–BII features. Of the three models built by *AmberTools*14, the models labelled as ‘Langridge’ and ‘Arnott’ fit the canonical BI BB00 class, but the dihedral angle values of ‘Langridge’ in particular are mostly found in the tail regions of the torsional distributions as defined in the golden set for BB00. The third model, ‘the average’, differs significantly from any major B-DNA conformer and is only weakly related to the rather exotic BB15, with a high α_1_ value and γ_1_ near 0°.

The labelling of several fibre models by codes suggesting the existence of distinct DNA conformations as C, D or B′ is not supported at the level of their backbone conformations because they have backbone structures classified by the same NtC classes. On the other hand, some other fibre models are labelled by the same code but are described by different NtC. For instance, ‘C-DNA’ models were found to have their backbones described by the canonical BB00 or by BII BB07; one duplex labelled as B′-DNA has one backbone described by BB07 and the other described by AA00.

### 3.6. Models based on the superposition of structures of NtC classes used as building blocks   

**Figure 8:**
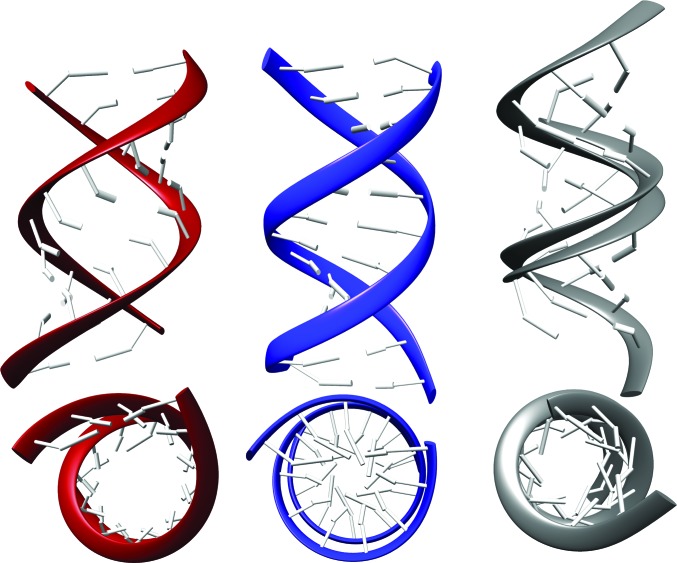
Models of A-DNA (red), BI-DNA (blue) and hypothetical BII-DNA (grey) based on the superposition of building blocks formed by base-paired dinucleotides with structures of NtC classes AA00, BB00 and BB07, respectively. The top images are rotated to show the minor groove in the middle of the duplexes; the bottom images are viewed along the helical axes.

We superimposed Watson–Crick paired steps in the conformations of NtC AA00, BB00 and BB07 to test whether these building blocks can potentially put together a duplex DNA. The models built from AA00 and BB00 display the main characteristics expected for A-DNA and B-DNA duplexes despite the fact that the models were built by simple superposition of the backbone atoms without any geometry or energy optimization (Fig. 8 ▸). This leads us to the conviction that the NtC classes can be used to build realistic models of DNA duplexes. In contrast, a hypothetical duplex composed solely of the BII NtC class BB07 cannot exist as its minor groove is too narrow and the phosphate groups on the opposing strands clash. Indeed, our data set of nearly 60 000 steps shows only 31 cases when two BB07 steps overlap in sequence, and in only three cases three BB07 steps overlap forming a tetranucleotide stretch in the BII-DNA form; no longer stretches exist.

## 4. Conclusions   

Our analysis of DNA structures led to the identification of 44 DNA step conformer classes called NtC (Supplementary Table S1). Structurally similar NtC classes were grouped into 11 letters of the DNA structural alphabet CANA (Table 1 ▸, Fig. 3 ▸), which makes the analysis of DNA structure more comprehensible yet does not compromise the impartiality of the structural description and provides a tool to characterize the DNA structure beyond a rough classification into BI-, BII-, A- and Z-DNA types. Annotation of the conformational properties of a few archetypal types of DNA structures revealed some unexpected features. The Dickerson–Drew dodecamer, which is often considered to be a typical B-DNA duplex, is conformationally rich, with a high proportion of features mixing B and A forms. Our analysis of duplex models based on the fibre-diffraction data discloses the need for their critical evaluation before they are used for computer modelling (Fig. 7 ▸, Supplementary Fig. S4). Conformational analysis of guanine quadruplexes demonstrates the universality of the most frequent B conformer, BB00, which builds the tetrad cores of these folded DNA in combination with more exotic conformers such as BBS1 or BB1S.

The ∼21% of steps that are left unassigned in our procedure represent a compromise between the accuracy of the assignment and the complex nature of the DNA conformational space: a relaxed accuracy of the assignment criteria can lead to an arbitrarily higher percentage of assigned conformers, but the broad overlapping torsion distributions would lead to increasing uncertainty of the assignment. A small percentage of the currently unassigned steps may subsequently be classified as new NtC classes. These classes may be quite important for understanding the detailed architecture of folded DNA, such as turns in hairpin structures or quadruplexes and still uncharacterized conformer(s) describing the i-motif fold, but they will most likely be numerically small. Undoubtedly, a fair number of the unassigned steps originate from refinement errors, but even error-free structures will have a significant number of uncharacterized conformers because of the high deformability of DNA molecules.

Both NtC classes and CANA letters are assigned automatically to any PDB-formatted DNA structure at the website https://dnatco.org (Černý *et al.*, 2016 ▸). A representation of the CANA letters embodied into the molecular graphics of DNA structures allows straightforward visual inspection of the main structural features of the DNA (Fig. 6 ▸). The definition of the NtC classes allowed us to define a validation score, confal, that quantifies the agreement between the conformation of an analyzed structure and the known NtC classes.

We believe that the NtC and CANA assignment protocol will contribute to understanding DNA structures by their impartial characterization, help to refine and validate DNA crystal and NMR structures, interpret DNA molecular modelling, and facilitate challenging analyses of sequence-dependent features of DNA structures and their interactions with proteins.

## 5. Related literature   

The following references are cited in the Supporting Information for this article: Adhireksan *et al.* (2014 ▸), Ahlrichs *et al.* (1989 ▸), Aymami *et al.* (1999 ▸), Boer *et al.* (2006 ▸), Davis *et al.* (2007 ▸), Frouws *et al.* (2016 ▸), Glas *et al.* (2009 ▸), Gleghorn *et al.* (2008 ▸), Goedecke *et al.* (2001 ▸), Johnson & Beese (2004 ▸), Jurečka *et al.* (2007 ▸), Kondo *et al.* (2014 ▸), Larkin *et al.* (2007 ▸), Mondragón & Harrison (1991 ▸), Nicoludis *et al.* (2012 ▸), Patikoglou *et al.* (1999 ▸), Schäfer *et al.* (1994 ▸), Schellenberg *et al.* (2012 ▸), Sriram *et al.* (1992 ▸), Swan *et al.* (2009 ▸), Tao *et al.* (2003 ▸), Temperini *et al.* (2003 ▸) and Williams *et al.* (2008 ▸).

## Supplementary Material

Supplementary text, tables and figures.. DOI: 10.1107/S2059798318000050/rr5151sup1.pdf

## Figures and Tables

**Table 1:** The 12 letters of the CANA DNA structural alphabet based on the NtC step-conformer classes and their presence in the 58 069 analyzed steps A brief annotation and other characteristics of all 44 NtC classes are given in Supplementary Table S1.

		Protein–DNA		Naked DNA		
The main features of the CANA letters	CANA letter	No.	%	No.	%	NtC classes merged into the CANA letter
A-form conformers	AAA	1942	3.8	1063	14.6	AA00 + AA01 + AA02 + AA03 + AA04
Conformers bridging A-form to B-form	A-B	2393	4.7	368	5	AB01 + AB02 + AB03
Conformers bridging B-form to A-form	B-A	3530	7	616	8.4	BA01–BA10, BA13–BA17
The most frequent ‘canonical’ B-form	BBB	17513	34.5	1561	21.4	BB00
Less populated BI conformer	2B1	3076	6.1	486	6.7	BB01
Less populated BI conformers with switched α_1_/γ_1_ values	3B1	2696	5.3	52	0.7	BB02 + BB03
Conformers bridging BI-form to BII-form	B12	3286	6.5	213	2.9	BB04 + BB05
BII conformers	BB2	2521	5	603	8.3	BB07 + BB08
Various minor B conformers	miB	2610	5.1	192	2.6	BB10–BB16
Conformers with bases in *syn* orientation, may occur in quadruplexes and other nonduplexes	SQX	214	0.4	248	3.4	AB1S + BBS1 + BB1S + BB2S + NS1S + NS02 + NS03 + NS04 + NS05
Z-forms	ZZZ	154	0.3	175	2.4	ZZ1S + ZZ2S + ZZS1 + ZZS2
All assigned steps		39935	78.7	5577	76.4	
Non-assigned steps	NAN	10839	21.3	1718	23.6	
All steps		50774	100.0	7295	100.0	
