# Supplementary material for: A DNA structural alphabet provides new insight into DNA flexibility
Source: Acta Crystallogr D Struct Biol. 2018 Jan 1;74(Pt 1):52–64. doi: 10.1107/S2059798318000050 (PMC5786007; doi:10.1107/S2059798318000050)
Supplement: Supplementary file 1 [file d-74-00052-sup1.pdf]

Acta Cryst D STRUCTURAL BIOLOGY

**Volume 74 (2018)**

**Supporting information for article:**

# A DNA structural alphabet provides a new insight into DNA flexibility

**Bohdan Schneider, Paulína Božíková, Iva Nečasová, Petr Čech, Daniel Svozil and Jiří Černý**

## ***Methods***

### *Retrieval of sequentially non-redundant structures*

*Selection of sequentially non-redundant set of structures*. To avoid using sequentially redundant structural data and reduce statistical bias, we compared structures sequentially. Before the sequential alignment, we excluded N-terminal and C-terminal tags, which were presumed not to interact with DNA. ClustalX (Larkin *et al.*, 2007) was used to divide protein sequences into two groups, sequentially unique and sequentially redundant. Proteins were labeled as identical in case of their 100% sequence identity. In the next step, we compared DNA sequences in complexes containing proteins marked as sequentially redundant and those with differed DNA sequences were kept. Several complexes with the same or similar protein sequence but different DNAs provide unique protein-DNA interfaces and contribute to the dataset by structural information.

DNA sequences were termed sequentially unique when their sequences shorter than 24 nucleotides differed by at least two nucleotides and sequences longer than 24 nucleotides sequences by at least 3 nucleotides. DNA sequences in structures of the histone core particle were aligned and sorted into groups, in which DNA differed by less than 11 nucleotides. There were nine such groups and in each, we selected one structure deemed the best. The best structure in a group of structures tagged as sequentially redundant was selected based on crystallographic resolution or MolProbity Score (Davis *et al.*, 2007): a structure was selected when its crystallographic resolution was better than in the redundant group by at least 0.2 Å, in case a few structures tied, the best was selected based on the MolProbity Score (Davis *et al.*, 2007).

***Methods*** : Enhancement of the original k-NN protocol and discovery of new ***NtC*** classes.

**Table S1**. Definitions of the 44 identified ***NtC*** conformer classes: a) ***NtC*** annotations, the averages of torsion angle values, and occurrences of ***NtC*** in the 1,802 analyzed structures; b) estimated standard deviations of the torsion averages of ***NtC***. PDB formatted coordinates of representative examples of all ***NtC*** can be found in a separate zip file and at dnatco.org.

| Annotation | ***CANA*** | ***NtC*** | 1 δ | 2 ε | 3 ζ | 4 α1 | 5 β1 | 6 γ1 | 7 δ1 | 8 χ | 9 χ1 | Numbers of Steps in prot/DNA # | prot/DNA % | naked DNA # | naked DNA % | golden set |
|---|---|---|---|---|---|---|---|---|---|---|---|---|---|---|---|---|
| the most frequent, "canonical" A-DNA (identical to A-RNA) | *AAA* | *AA00* | 83 | 206 | 287 | 293 | 174 | 55 | 83 | 199 | 202 | 1 173 | 2.3 | 866 | 11.9 | 296 |
| A-DNA with α1/γ1 crank (150/180) | *AAA* | *AA01* | 82 | 194 | 291 | 149 | 193 | 182 | 87 | 205 | 188 | 163 | 0.3 | 88 | 1.2 | 55 |
| A-DNA with BI-like χ/χ1 | *AAA* | *AA02* | 88 | 203 | 275 | 294 | 161 | 54 | 88 | 244 | 245 | 554 | 1.1 | 63 | 0.9 | 168 |
| A-DNA similar to AA01, high α1 | *AAA* | *AA03* | 83 | 214 | 273 | 330 | 165 | 26 | 86 | 202 | 215 | 16 | 0.0 | 14 | 0.2 | 9 |
| A-DNA similar to AA01, low α1 | *AAA* | *AA04* | 80 | 198 | 301 | 255 | 175 | 91 | 83 | 202 | 192 | 36 | 0.1 | 32 | 0.4 | 14 |
| A-to-B: δ C3'-endo, δ1 C2'-endo, χ/χ1 have A/B pattern | *A-B* | *AB01* | 86 | 193 | 283 | 299 | 180 | 55 | 142 | 221 | 255 | 2 190 | 4.3 | 347 | 4.8 | 100 |
| A-to-B: δ ~ O4'-endo, extremely low ε, χ/χ1 are B-like | *A-B* | *AB02* | 93 | 59 | 56 | 208 | 187 | 65 | 133 | 240 | 252 | 116 | 0.2 | 1 | 0.0 | 18 |
| A-to-B: similar to AB01 with high α1 and low ζ, β1, γ1 | *A-B* | *AB03* | 96 | 195 | 253 | 344 | 151 | 33 | 131 | 220 | 250 | 87 | 0.2 | 20 | 0.3 | 11 |
| BI-to-A complex conformer with δ1 from C3' to C4'-exo, χ/χ1 are B-like | *B-A* | *BA01* | 134 | 189 | 258 | 293 | 169 | 51 | 88 | 248 | 229 | 749 | 1.5 | 142 | 1.9 | 54 |
| BI-to-A complex conformer with δ1 from C3' to O4'-endo, χ/χ1 are B-like | *B-A* | *BA05* | 130 | 184 | 266 | 295 | 170 | 53 | 102 | 249 | 235 | 2083 | 4.1 | 394 | 5.4 | 107 |
| B-to-A: β1~120, χ1 A-like | *B-A* | *BA08* | 141 | 212 | 188 | 302 | 129 | 55 | 85 | 271 | 211 | 92 | 0.2 | 34 | 0.5 | 12 |
| BI-to-A: β1~60, α1/γ1 crank (250/170), χ1 A-like | *B-A* | *BA09* | 135 | 199 | 287 | 254 | 71 | 168 | 86 | 265 | 187 | 133 | 0.3 | 3 | 0.0 | 13 |
| B-to-A complex cluster: α1~100, γ1~180, χ1 A-like | *B-A* | *BA10* | 135 | 200 | 218 | 105 | 229 | 194 | 88 | 258 | 195 | 203 | 0.4 | 6 | 0.1 | 43 |
| BII-to-A complex cluster: α1~60, γ1~180, χ1 A-like | *B-A* | *BA13* | 142 | 231 | 196 | 74 | 232 | 196 | 89 | 266 | 199 | 153 | 0.3 | 8 | 0.1 | 57 |
| BII-to-A: χ1 A-like | *B-A* | *BA16* | 145 | 255 | 187 | 63 | 226 | 196 | 87 | 260 | 201 | 11 | 0.0 | 1 | 0.0 | 7 |
| BII-to-A: δ1~O4'-endo, β1~120 | *B-A* | *BA17* | 148 | 255 | 177 | 294 | 130 | 44 | 96 | 272 | 234 | 106 | 0.2 | 28 | 0.4 | 27 |
| the most frequent DNA conformer, "canonical" B form, also called BI | ***BBB*** | ***BB00*** | **138** | **183** | **259** | **303** | **180** | **44** | **138** | **252** | **258** | 17 513 | 34.5 | 1 561 | 21.4 | 1 958 |
| less populated variant of BI form | *2B1* | *BB01* | 132 | 181 | 265 | 301 | 177 | 49 | 121 | 248 | 244 | 3 076 | 6.1 | 486 | 6.7 | 142 |
| BI with α1/γ1 crank (30/300) | *3B1* | *BB02* | 140 | 194 | 246 | 32 | 194 | 297 | 150 | 252 | 253 | 2 369 | 4.7 | 47 | 0.6 | 134 |
| BI with α1/γ1 crank (170/170) | *3B1* | *BB03* | 145 | 176 | 276 | 166 | 164 | 173 | 146 | 239 | 232 | 327 | 0.6 | 5 | 0.1 | 31 |
| BI-to-BII conformer | *B12* | *BB04* | 140 | 201 | 214 | 315 | 153 | 46 | 140 | 263 | 253 | 3 179 | 6.3 | 211 | 2.9 | 243 |
| BI-to-BII with α1/γ1 crank (70/230), χ1 A-like | *B12* | *BB05* | 143 | 219 | 202 | 68 | 232 | 220 | 124 | 269 | 209 | 107 | 0.2 | 2 | 0.0 | 20 |
| BII form, typical by ε/ζ switch compared to BI | *BB2* | *BB07* | 144 | 245 | 171 | 297 | 141 | 46 | 141 | 270 | 259 | 2 415 | 4.8 | 598 | 8.2 | 371 |
| BII form, variant, α1/γ1 crank (60/210) | *BB2* | *BB08* | 144 | 249 | 195 | 63 | 229 | 211 | 144 | 261 | 231 | 106 | 0.2 | 5 | 0.1 | 19 |
| B form with extremely low values of α1, β1, γ1 | *miB* | *BB10* | 139 | 195 | 191 | 23 | 106 | 19 | 129 | 257 | 258 | 877 | 1.7 | 15 | 0.2 | 49 |
| B form with α1/γ1 crank (120/180) | *miB* | *BB11* | 144 | 200 | 199 | 121 | 226 | 189 | 143 | 258 | 222 | 238 | 0.5 | 2 | 0.0 | 22 |
| BI with α1/γ1 crank (250/170), low β1 70, χ1 A-like | *miB* | *BB12* | 140 | 196 | 287 | 248 | 73 | 172 | 144 | 263 | 212 | 414 | 0.8 | 3 | 0.0 | 18 |
| BI with α1/γ1 crank (210/160), low β1 100, χ1 A-like | *miB* | *BB13* | 143 | 186 | 291 | 216 | 104 | 161 | 147 | 252 | 219 | 179 | 0.4 | 3 | 0.0 | 17 |
| B-form with extremely low ε | *miB* | *BB14* | 121 | 104 | 303 | 229 | 260 | 73 | 132 | 264 | 263 | 109 | 0.2 | 0 | 0.0 | 8 |
| BI with high α1 and γ1 near 0 | *miB* | *BB15* | 149 | 186 | 262 | 340 | 194 | 354 | 149 | 251 | 262 | 313 | 0.6 | 31 | 0.4 | 17 |
| complex cluster of B-like conformers: A-like χ, bases may be unstacked to incorporate intercalated drug, occurs where backbone accommodates deformation (metal ion near, strand crossing in Holliday junctions, ends of duplexes) | *miB* | *BB16* | 145 | 227 | 281 | 288 | 173 | 51 | 142 | 197 | 267 | 480 | 0.9 | 138 | 1.9 | 117 |
| A-to-B-like but χ1 syn, mostly in duplexes | *SQX* | *AB1S* | 91 | 214 | 280 | 295 | 176 | 56 | 139 | 238 | 67 | 17 | 0.0 | 0 | 0.0 | 14 |
| BI-like but χ syn, may be in duplex but many G-G in quadruplexes | *SQX* | *BBS1* | 146 | 189 | 275 | 294 | 174 | 52 | 135 | 62 | 261 | 145 | 0.3 | 158 | 2.2 | 47 |
| G-G in quadruplexes, unusual β1, γ1, χ1 syn | *SQX* | *BB1S* | 140 | 202 | 282 | 307 | 258 | 304 | 151 | 236 | 65 | 4 | 0.0 | *21* | 0.3 | 17 |
| BI-like but χ1 syn, mostly G-G in quadruplexes, α1 g+, γ1 g- | *SQX* | *BB2S* | 137 | 196 | 225 | 33 | 187 | 295 | 145 | 257 | 70 | 7 | 0.0 | *8* | 0.1 | 8 |
| part. unstacked T-G/G-T in quadruplexes, χ1 syn, unusual ζ, α1, γ1 | *SQX* | *NS1S* | 143 | 206 | 61 | 82 | 204 | 192 | 146 | 242 | 68 | 5 | 0.0 | *15* | 0.2 | 13 |
| start of loop in quadruplex or hairpin, G-X, untypical ζ, α1, γ1 | *SQX* | *NS02* | 145 | 225 | 67 | 74 | 189 | 191 | 137 | 264 | 258 | 11 | 0.0 | *21* | 0.3 | 14 |
| part. unstacked T-T in quadruplexes, unusual combination of ε, ζ, α1, γ1, χ1 | *SQX* | *NS03* | 143 | 294 | 111 | 153 | 197 | 53 | 151 | 262 | 185 | 5 | 0.0 | *11* | 0.2 | 13 |
| unstacked, in 4-way junction, ε, ζ high values | *SQX* | *NS04* | 140 | 275 | 279 | 304 | 191 | 56 | 150 | 266 | 210 | 2 | 0.0 | *14* | 0.2 | 12 |
| 5'-end of dsDNA, base open (unstacked), ζ, α1 ~60 | *SQX* | *NS05* | 154 | 242 | 77 | 63 | 177 | 64 | 137 | 237 | 249 | 18 | 0.0 | *0* | 0.0 | 10 |
| Z form, Y-R step | *ZZZ* | *ZZ1S* | 148 | 264 | 76 | 66 | 186 | 179 | 95 | 205 | 60 | 65 | 0.1 | 73 | 1.0 | 20 |
| Z form, Y-R step, δ1 C2'-endo | *ZZZ* | *ZZ2S* | 141 | 263 | 71 | 78 | 179 | 185 | 148 | 208 | 77 | 33 | 0.1 | 38 | 0.5 | 41 |
| ZI form, R-Y step | *ZZZ* | *ZZS1* | 97 | 242 | 294 | 209 | 230 | 55 | 144 | 63 | 205 | 49 | 0.1 | 52 | 0.7 | 48 |
| ZII form, R-Y step | *ZZZ* | *ZZS2* | 95 | 186 | 63 | 169 | 162 | 44 | 144 | 58 | 213 | 7 | 0.0 | 12 | 0.2 | 17 |
| Unassigned conformations | *NAN* | *NANT* | × | × | × | × | × | × | × | × | × | 10 839 | 21.3 | 1 718 | 23.6 | 0 |
| | | | δ | ε | ζ | α1 | β1 | γ1 | δ1 | χ | χ1 | **50 774** | **100.0** | **7 295** | **100.0** | **4 431** |
| Annotation | ***CANA*** | ***NtC*** | 1 | 2 | 3 | 4 | 5 | 6 | 7 | 8 | 9 | # prot/DNA | % | # naked | % | golden set |

# **Table S1**. b) estimated standard deviations of the torsion averages of ***NtC*** .

| | | 1 | 2 | 3 | 4 | 5 | 6 | 7 | 8 | 9 | ***Quality of Golden set (*)*** | |
|---|---|---|---|---|---|---|---|---|---|---|---|---|
| ***CANA*** | ***NtC*** | esd(δ) | esd(ε) | esd(ζ) | esd(α1) | esd(β1) | esd(γ1) | esd(δ1) | esd(χ) | esd(χ1) | rmsd of 9 torsions [°] | cartesian rmsd [Å] |
| *AAA* | *AA00* | 4.8 | 7.7 | 5.9 | 6.2 | 8.0 | 6.1 | 4.4 | 7.3 | 6.9 | 13.3 | 0.45 |
| *AAA* | *AA01* | 3.7 | 7.6 | 5.9 | 8.3 | 6.6 | 5.8 | 3.7 | 4.7 | 4.6 | 12.0 | 0.33 |
| *AAA* | *AA02* | 5.9 | 8.9 | 9.9 | 6.9 | 8.2 | 4.9 | 5.5 | 7.8 | 7.4 | 12.7 | 0.63 |
| *AAA* | *AA03* | 6.2 | 5.9 | 4.3 | 4.4 | 6.3 | 7.1 | 5.6 | 8.7 | 7.0 | 8.9 | 0.33 |
| *AAA* | *AA04* | 8.7 | 9.3 | 6.1 | 6.1 | 10.6 | 6.2 | 7.2 | 7.2 | 7.9 | 11.3 | 0.39 |
| *A-B* | *AB01* | 3.8 | 12.6 | 7.9 | 7.7 | 7.9 | 6.0 | 4.4 | 11.9 | 8.6 | 15.5 | 0.53 |
| *A-B* | *AB02* | 8.7 | 8.9 | 9.1 | 11.8 | 9.5 | 8.0 | 7.9 | 10.4 | 8.1 | 13.1 | 0.43 |
| *A-B* | *AB03* | 7.8 | 6.2 | 6.3 | 6.2 | 7.7 | 7.2 | 9.1 | 3.6 | 6.5 | 8.8 | 0.44 |
| *B-A* | *BA01* | 6.8 | 4.4 | 7.8 | 10.6 | 12.3 | 4.6 | 3.3 | 11.1 | 6.7 | 6.0 | 0.16 |
| *B-A* | *BA05* | 8.4 | 7.6 | 7.0 | 7.6 | 5.5 | 7.2 | 7.8 | 8.7 | 7.4 | 14.1 | 0.42 |
| *B-A* | *BA08* | 6.6 | 6.8 | 4.9 | 4.8 | 6.0 | 5.8 | 5.5 | 5.5 | 3.1 | 13.1 | 0.50 |
| *B-A* | *BA09* | 4.4 | 5.7 | 5.2 | 5.7 | 10.0 | 6.0 | 4.6 | 4.7 | 4.7 | 9.9 | 0.27 |
| *B-A* | *BA10* | 10.7 | 10.2 | 20.1 | 19.6 | 16.1 | 12.3 | 6.4 | 12.3 | 6.5 | 7.6 | 0.26 |
| *B-A* | *BA13* | 6.1 | 11.7 | 11.5 | 10.6 | 8.3 | 10.1 | 6.9 | 8.5 | 7.0 | 20.4 | 0.65 |
| *B-A* | *BA16* | 5.1 | 11.4 | 4.4 | 8.2 | 6.9 | 2.8 | 3.2 | 3.3 | 3.2 | 14.0 | 0.43 |
| *B-A* | *BA17* | 8.1 | 8.6 | 10.1 | 9.5 | 9.0 | 7.4 | 7.8 | 9.4 | 8.1 | 8.8 | 0.30 |
| ***BBB*** | ***BB00*** | **5.8** | **9.3** | **10.2** | **9.2** | **10.5** | **6.0** | **5.1** | **9.0** | **9.9** | **12.0** | **0.52** |
| *2B1* | *BB01* | 8.1 | 6.9 | 6.0 | 7.7 | 5.6 | 6.4 | 3.8 | 8.6 | 6.0 | 16.8 | 0.80 |
| *3B1* | *BB02* | 5.6 | 7.0 | 7.8 | 8.8 | 14.2 | 8.5 | 4.8 | 8.9 | 9.3 | 10.3 | 0.43 |
| *3B1* | *BB03* | 5.6 | 7.4 | 8.2 | 14.5 | 12.1 | 8.6 | 6.5 | 12.1 | 9.1 | 15.8 | 0.47 |
| *B12* | *BB04* | 4.4 | 9.5 | 9.0 | 8.1 | 9.0 | 6.4 | 4.5 | 8.9 | 8.6 | 14.5 | 0.57 |
| *B12* | *BB05* | 6.0 | 7.8 | 13.4 | 10.8 | 7.9 | 12.4 | 8.0 | 10.6 | 9.5 | 12.2 | 0.44 |
| *BB2* | *BB07* | 5.1 | 12.3 | 9.6 | 8.8 | 7.6 | 6.0 | 5.5 | 9.7 | 11.2 | 13.8 | 0.35 |
| *BB2* | *BB08* | 7.5 | 13.7 | 13.0 | 7.4 | 9.9 | 8.3 | 9.3 | 10.0 | 9.2 | 15.3 | 0.50 |
| *miB* | *BB10* | 5.2 | 10.3 | 9.2 | 11.4 | 9.0 | 9.6 | 7.5 | 10.3 | 9.6 | 13.2 | 0.47 |
| *miB* | *BB11* | 6.2 | 9.6 | 9.0 | 12.5 | 11.3 | 10.2 | 7.0 | 10.0 | 9.9 | 12.8 | 0.46 |
| *miB* | *BB12* | 4.6 | 6.0 | 11.7 | 13.7 | 3.8 | 4.2 | 4.4 | 8.0 | 9.0 | 13.5 | 0.52 |
| *miB* | *BB13* | 7.3 | 7.9 | 11.4 | 9.4 | 10.5 | 7.0 | 7.9 | 7.9 | 7.5 | 11.5 | 0.43 |
| *miB* | *BB14* | 6.0 | 6.8 | 5.6 | 7.2 | 4.2 | 8.6 | 4.6 | 8.7 | 5.9 | 11.9 | 0.46 |
| *miB* | *BB15* | 4.9 | 6.0 | 10.1 | 10.2 | 10.0 | 12.9 | 5.0 | 9.6 | 7.2 | 6.9 | 0.35 |
| *miB* | *BB16* | 7.4 | 12.8 | 11.2 | 8.7 | 10.8 | 8.0 | 5.3 | 9.3 | 18.4 | 13.3 | 0.44 |
| *SOX* | *AB1S* | 2.4 | 3.3 | 4.4 | 4.6 | 4.4 | 3.6 | 4.9 | 4.2 | 3.7 | 16.0 | 0.73 |
| *SOX* | *BBS1* | 5.1 | 8.7 | 7.7 | 9.6 | 6.9 | 7.7 | 6.8 | 9.4 | 10.8 | 9.2 | 0.30 |
| *SOX* | *BB1S* | 6.6 | 9.3 | 6.8 | 5.6 | 6.7 | 4.9 | 3.5 | 12.7 | 3.9 | 5.5 | 0.17 |
| *SOX* | *BB2S* | 2.6 | 3.1 | 2.3 | 6.4 | 3.7 | 3.1 | 1.9 | 4.8 | 2.6 | 14.2 | 0.45 |
| *SOX* | *NS1S* | 2.5 | 6.3 | 7.0 | 7.9 | 6.0 | 6.1 | 4.8 | 4.5 | 4.7 | 12.2 | 0.80 |
| *SOX* | *NS02* | 2.8 | 9.3 | 6.6 | 12.1 | 7.9 | 7.7 | 9.1 | 12.5 | 8.3 | 8.1 | 0.22 |
| *SOX* | *NS03* | 3.2 | 3.9 | 6.2 | 8.5 | 4.7 | 6.4 | 4.4 | 5.9 | 2.3 | 8.7 | 0.28 |
| *SOX* | *NS04* | 3.5 | 6.2 | 7.3 | 9.3 | 7.9 | 7.1 | 2.6 | 6.4 | 3.9 | 6.7 | 0.13 |
| *SOX* | *NS05* | 2.1 | 2.0 | 4.0 | 5.3 | 2.2 | 4.2 | 1.3 | 4.7 | 3.1 | 7.5 | 0.27 |
| *ZZZ* | *ZZ1S* | 3.5 | 5.3 | 4.0 | 3.8 | 4.4 | 3.5 | 5.0 | 5.5 | 6.1 | 7.0 | 0.18 |
| *ZZZ* | *ZZ2S* | 4.5 | 5.5 | 6.2 | 7.0 | 6.7 | 4.7 | 6.6 | 6.8 | 4.7 | 10.1 | 0.30 |
| *ZZZ* | *ZZS1* | 5.8 | 7.2 | 8.7 | 6.9 | 10.1 | 5.8 | 4.9 | 4.9 | 6.4 | 12.3 | 0.31 |
| *ZZZ* | *ZZS2* | 2.0 | 5.6 | 6.4 | 6.0 | 6.6 | 5.1 | 2.9 | 4.3 | 4.6 | 6.8 | 0.19 |
| *NAN* | *NANT* | × | × | × | × | × | × | × | × | × | | |
| ***CANA*** | ***NtC*** | esd(δ) | esd(ε) | esd(ζ) | esd(α1) | esd(β1) | esd(γ1) | esd(δ1) | esd( χ) | esd( χ1) | | |
| | | 1 | 2 | 3 | 4 | 5 | 6 | 7 | 8 | 9 | | |

*(*) "rmsd of 9 torsions" is rmsd measured in torsion space between the mean and most distant member of the golden set; "cartesian rmsd" is rmsd between 18 atoms of the dinucleotide that define the nine torsions*

*Conformational assignment of a dinucleotide*

*Algorithm assigning the **NtC** conformational classes* is schematically shown in a flowchart in Figure S1. The assignment process is summarized below.

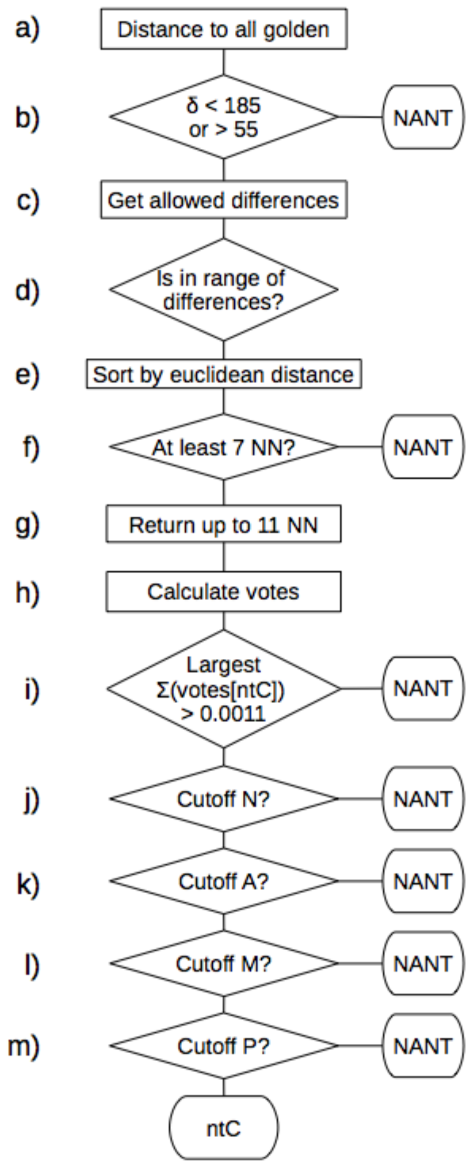


**Figure S1**. Schema of the algorithm assigning ***NtC*** classes to DNA dinucleotides.

a) In the initial step, distances are calculated between candidate dinucleotide and all dinucleotides in the golden set. All the distance, angular average and estimated standard deviation (ESD) formulas take into account the circularity (0-360°) of the data.

b) Steps with delta torsions outside the physically possible region are marked as unassigned conformer (NANT).

c) The allowed difference from the average is calculated as 5 times the ESD of the respective torsion within the known ***NtC*** class (ESD and class average are calculated for the golden set using functions from the R package http://www.R-project.org/).

d) The distances between the to-be-assigned dinucleotide and the golden set members are then tested if they are within the allowed limit.

e) Sort the ***NtC*** classes satisfying condition d) by their Euclidean distances from the to-be-assigned dinucleotide.

f) First up to 11 nearest neighbors are stored.

g) At least 7 neighbors are necessary to assign a ***NtC*** conformer.

h) For each ***NtC*** class a sum of votes is calculated (as 1/(Euclidean distance)^2).

i) If the maximal sum exceeds 0.0011 (1/(30^2)), the step is a candidate for the conformer class, subject to the following tests for final classification.

j) Each torsion must lie within 28º cutoff of a corresponding torsion of the nearest neighbor.

k) Each torsion must lie within 28º cutoff of an average of corresponding torsion over all nearest neighbors (7 to 11 neighbors).

l) The sum of (signed) differences from the candidate cluster average for first seven torsions ($\delta$ to $\delta 1$) as well as sum of torsions 8 and 9 (both $\chi$ torsions) must lie within ±60º. The value of ±60º was selected based on the analysis of sums of torsions from the data assigned without this test (Figure S3) in order to minimize false assignment of steps with systematically shifted torsion angles.

*Verification of dinucleotide membership to the golden set.* The data in the *golden set* were verified for self-consistency by comparing each member of the original *golden set* to the rest; ESD and averages of remaining data were calculated on the fly. If the step was assigned to a different ***NtC*** class than to the class it obtained in the original *golden set*, it was removed. The procedure was repeated until self-consistent golden set was obtained. Both cutoffs used in steps j) and k) above were set to a more stringent value of 20º for this purpose.

To summarize, the definition of the ***NtC*** classes and the algorithm of their assignment is not a result of a standard statistical procedure. All nine dinucleotide torsions have non-normal overlapping distributions that must be analyzed simultaneously in a complex 9-D space using noisy experimental data. The data are often of limited quality and quantity, even when we make use of virtually the complete set of non-redundant DNA structures available at the date of retrieval. The complexity of the 9-D torsional space required combining formal mathematical and statistical tools with knowledge-based empirical rules and involved human intervention at several critical steps.

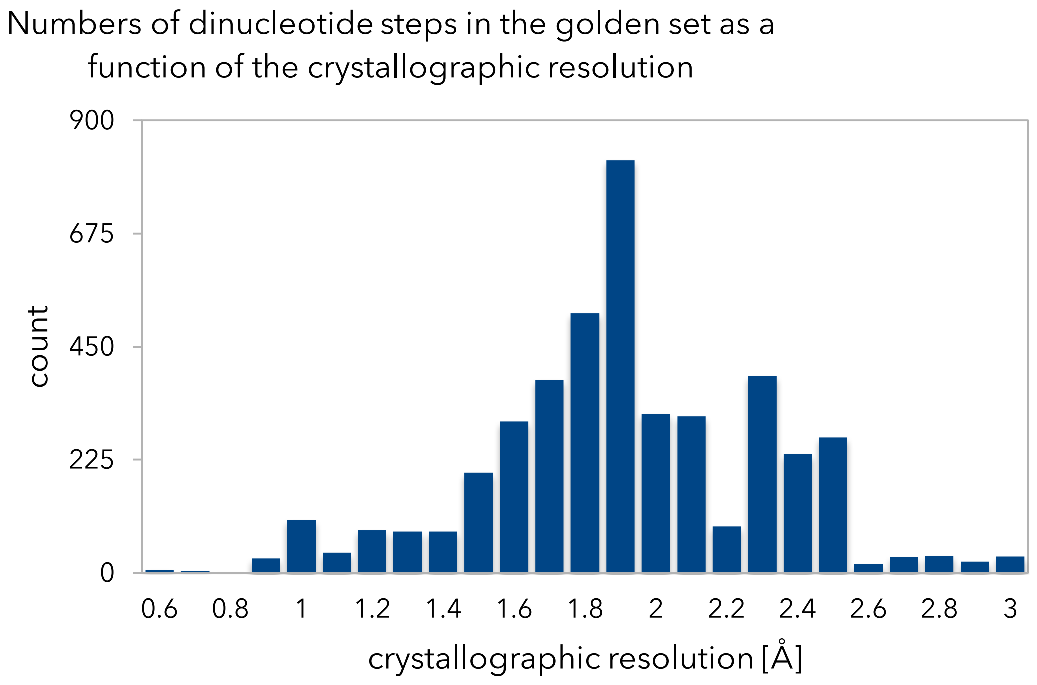


**Figure S2**. Histogram of numbers of dinucleotide steps in the *golden set* as a function of crystallographic resolution.

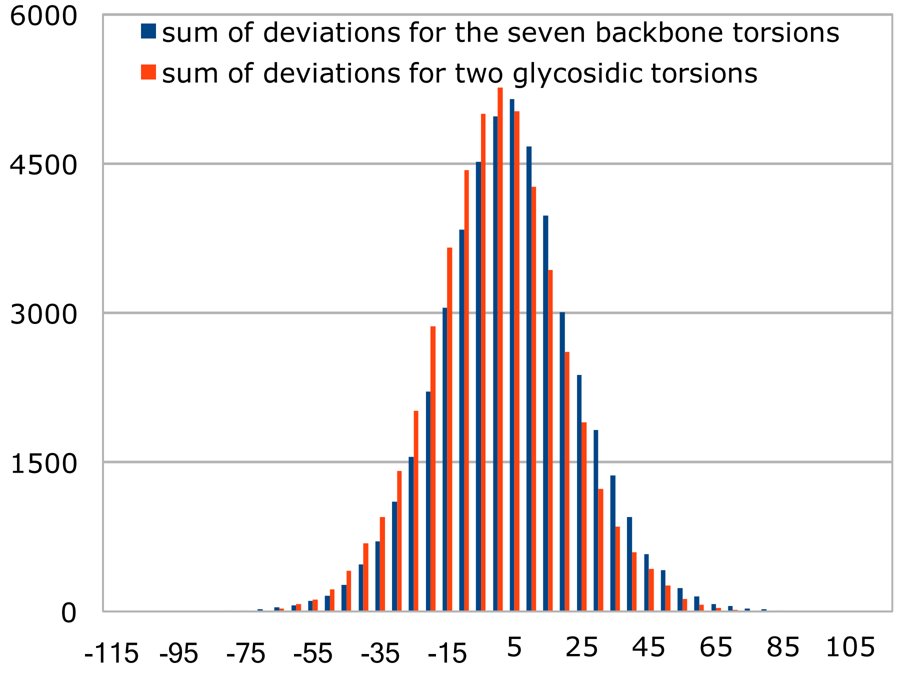


**Figure S3**. Histogram of sums of differences between the average of a particular ***NtC*** and the actual torsion values for dinucleotides assigned to this ***NtC*** in the *golden set*. Blue bars show differences of the backbone torsions δ, ε, ζ, α1, β1, γ1, δ1, the red ones of both glycosidic torsions χ and χ1. Both distributions are Gaussian and have about the same spread. Numbers of dinucleotides with deviations larger than ±60º is negligible.

### *Results*

*AAA: A-form*. The main conformational feature of the A-form is the sugar pucker in the C3'-*endo* region and the corresponding δ torsion values 80°-90°. The most frequent A-DNA conformer described here as ***NtC** AA00* is also characterized by a low *anti* glycosidic torsion χ near 200°. It is structurally identical to the A-RNA. ***NtC** AA01* differs from "the canonical" *AA00* by a typical "crankshaft motion" switch of torsion angles α and γ from values 300°/60° to 150°/180°. *AA02* is close to the canonical *AA00*, but its glycosidic torsions χ have B-like values near 250°.

*A-B: conformers bridging A- to B-form*. The main A-B conformer, *AB01*, has been described previously (Svozil *et al.*, 2008) and is characterized by an A-like sugar pucker C3'-*endo* region (δ ~85°) and by values of the glycosidic torsion χ near 230° in its first (5'-) nucleotide, and B-like values of torsions α1, β1, γ1, δ1, and χ1 in the second nucleotide. *AB01* is complemented by a newly characterized conformer *AB02*. It is interesting by its extremely low value of torsion ε near 60°, which is compensated by a switch of the following torsion ζ from a typical value near 300° to 60°. Despite the exotic value of ε, *AB02* is observed relatively frequently, 164 times in more than 100 structures, mostly in double helices of naked or complexed DNA, but also in hairpin stems (3dsd_C_DC17_DT18 (Williams *et al.*, 2008)) and in unpaired strand ends (4gz2_C_DC1_DA2 (Schellenberg *et al.*, 2012)).

*Analysis of the stability of conformer AB02.* The unprecedentedly low value of ε torsion near 60º prompted us to investigate whether this conformation is energetically stable as an isolated molecule or whether it is stabilized by intermolecular interactions in the crystal phase. We therefore performed quantum mechanical calculations of the internal potential energy of this conformer by scanning the ε torsion of the dinucleotide DG105 - DG106 of chain P from the high-resolution structure 4fj7 (Xia *et al.*, 2013) between 30º and 80º. The results of the computations, which are detailed in Table S2 and the main text Figure 4, showed a near-harmonic potential with minimal energy near ε of 55º demonstrating that the *AB02* conformer is locally stable as an isolated molecule.

**Table S2**. Torsion values (in degrees) in the ***NtC** AB02*, a representative dinucleotide from structure 4fj7 (Xia *et al.*, 2013), and after quantum mechanical optimization by DFT.

| | δ | ε | ζ | α | β | γ | δ1 | χ | χ1 |
|---|---|---|---|---|---|---|---|---|---|
| *AB02* average | 92 | 61 | 59 | 208 | 185 | 64 | 133 | 237 | 253 |
| 4FJ7_P_DG105_DG106 | 91 | 60 | 48 | 209 | 189 | 68 | 136 | 242 | 251 |
| energy-optimized torsions | 83 | 55 | 69 | 210 | 174 | 61 | 129 | 242 | 251 |

The hydrogens were added using ***openbabel*** and their positions optimized while keeping all the positions of heavy atoms frozen resulting in a dinucleotide with the overall charge of -2 (-1 at each phosphate group). During the scans, the position of heavy atoms of both guanine bases and C1' atoms were forced frozen, while positions of the remaining heavy atoms of the sugar phosphate backbone and of all hydrogen atoms were optimized. The solvation effects

were included using the COSMO model with a value of 78.4 for the solvent permittivity. To compute intramolecular energy of the step, we used dispersion corrected DFT functional TPSS (Tao *et al.*, 2003) and TZVP (Schafer *et al.*, 1994) augmented with empirical dispersion correction (Jurecka *et al.*, 2007) employing the TurboMole package of programs (Ahlrichs *et al.*, 1989).

*B1A and B2A: conformers bridging B- to A-form*. B-to-A conformers represent a structural counterpart of the previous ***CANA*** letter, *A-B*. The *B1A* and *B2A* letters are however represented by a much more diverse and numerous group of conformers than the *A-B* letter. The eight B-A conformers have the first sugar in the C2'-*endo* pucker and ε/ζ combination typical of either BI (180°/260°, *B1A* letter) or BII conformation (250°/170°, *B2A*). The sugar puckers of the second nucleotide are either in a typical C3'-*endo* or, in minority, O4'-*endo* pucker with δ1 near 100°. Conformationally soft glycosidic torsions correlate with the sugar pucker as expected: while χ is B-like, χ1 is lower and more A-like. Some of the B-to-A conformers have less common combinations of torsions ζ, α1, β1, and γ1 including α1/γ1 crankshaft in *BA10* and *BA13*, or an extremely small value of β in *BA09*. All B-to-A conformers occur in duplexes but they can also occur in quadruplexes (*BA13* in 3sc8, 3sc8_A_DG14_DG15 (Collie *et al.*, 2012)) or joints of Holliday junctions (*BA05* in 1p4e, 1p4e_J_DT15_DT16 (Chen & Rice, 2003)).

*Conformers of the B-form.* What is usually described as "B-DNA form" should be seen as a family of conformations rather than a single, firmly defined structure. Neither do the two traditionally recognized B forms, BI and BII, suffice to describe the conformational diversity of the B-like backbone. In the structural alphabet ***CANA***, we describe the B-form by three letters for BI conformers, one letter for BII conformers, and one letter for transitional BI-to-BII conformers. In addition, we grouped numerically minor but structurally diverse conformers that still bear some features of the B-form, under the letter *miB*.

*BBB, 2B1, and 3B1: BI conformers*. The dominant DNA conformer is a BI ***NtC*** *BB00*. It alone constitutes letter *BBB* because it represents about one third of all analyzed steps and should be viewed as the canonical B-form. It has both sugars in the C2'-*endo* pucker, values of both glycosidic torsions χ high *anti* near 250°, and displays a characteristic pattern of ε, ζ, α1, β1, γ1: t/g-/g-/t/g+ (180°, 260°, 300°, 180°, 40°). The related BI conformer *BB01* represents a slight but distinct and highly populated variation of *BB00*. Less populated conformers *BB02* and *BB03* (***CANA*** *3B1*) are characterized by a switch of the α1/γ1 values from the prevailing 300°/40° to 30°/300° and 170°/170°, respectively.

Dinucleotides whose conformations belong to the letters *BBB*, *2B1*, and *3B1* form the core of the Watson-Crick-paired double helices, but they can occur in almost any structural context including quadruplexes, or joints of Holliday junctions. This adaptability to various local constraints further demonstrates the role of BI as the main B-DNA conformation.

*BB2 and B12: BII and BI-to-BII conformers*. The main characteristics telling apart BI and BII forms is a switch of values of ε and ζ torsions from 180°/260° in BI to about 250°/180° in BII. The most frequent conformer epitomizing BII form is *BB07*, also typical by a low value of torsion β1, 140°. The other BII conformer, *BB08*, in addition has α1/γ1 values switched to ~60°/210°. The main BI-to-BII conformer, *BB04*, has torsion angle values indeed half-way between those of BI *BB00* and BII *BB07*.

A compensatory switch of α1 and γ1 torsions from their most frequently observed combination of 300°/50° is a common local variation of the backbone. Observed are combinations (i) 30°/300° in highly populated ***NtC*** *BB02*; (ii) 60°/210° in BII conformer *BB08* and BI-to-BII *BB05*; (iii) 150°/180° in the "classical" crankshaft motion A-DNA modification in *AA01* and in BI *BB03*. In all these cases, the low α value correlates with β

well above the usual value of 180°.

*miB: various "minor" B-forms*. B-forms exist in several distinct conformers that share typical conformational features of the B-form: both sugars in the C2'-*endo* pucker and both glycosidic torsions χ above 200°. Some of these 'minor' forms are actually fairly common: *BB10* represents 1.6 %, *BB12* 0.8 %, and *BB16* 1.3 % of the analyzed steps. The structural features of these conformers can be quite exotic, but they do not disrupt base stacking and are compatible with incorporation into a double helix. Conformer *BB10* has extremely low values of α, β, γ: 20°/100°/20°, but it occurs quite frequently in both naked DNA (458d_A_DC1_DG2 (Aymami *et al.*, 1999)) and in ~300 protein complexes, e.g.3cro_A_DA5_DC6 (Mondragon & Harrison, 1991), and in 4kgc (4kgc_I_DA-31_DA-30 (Adhireksan *et al.*, 2014)), where it was detected 36 times. Even more extreme γ values near 0° are observed in *BB15*, but even this ***NtC*** does not disrupt the double helical architecture and participates in W-C pairing in naked DNA (3gqc_G_DT10_DA11 (Swan *et al.*, 2009)), DNA with a drug bound in the minor groove (netropsin in 1d86, 1d86_B_DT20_DC21 (Sriram *et al.*, 1992)), as well as in a protein-bound DNA (3lz1_J_DC18_DC19 (Vasudevan *et al.*, 2010)). *BB15* also occurs in the central region of guanine quadruplexes (1k8p_B_DG22_DG23 (Parkinson *et al.*, 2002)). *BB14* is similar to *AB02*, which was discussed above, by its extremely low value of ε torsion, and is fully compatible with the W-C pairing. Contrary to *AB02*, it is frequently found in nucleosomes.

***NtC*** *BB16* is a complex B-like conformer without any extreme torsion value, which is, however, important by the ability to accommodate a large local deformation of the strand. A particular combination of torsions attributable to this conformer can lead to unstacking of the bases to incorporate an intercalated drug (1nab_A_DC1_DG2 (Temperini *et al.*, 2003)), 308d_A_DC1_DG2(Gao *et al.*, 1997)), or to stack a base between two bases of the complementary strand (1g38_C_DT705_DC706 (Goedecke *et al.*, 2001)). *BB16* is also often found at or near strand crossing in Holliday junctions (1l4j_D_DC37_DC38 (Thorpe *et al.*, 2003)), near a metal cation (e.g. mutually close 3 $Mg^{++}$ in 1mow_B_DC312_DA313 (Chevalier *et al.*, 2002)), at bends of histone complexes (5f99_J_DA-70_DA-69 (Frouws *et al.*, 2016)), or in a hairpin loop (2cdm_B_DT23_DC24 (Boer *et al.*, 2006)).

*SQX: conformers occurring mainly in non-duplex DNA*. Conformers from this structurally variable group are rare but important for the architecture of folded forms of DNA, because dinucleotides in these conformations are found in unpaired or mismatched parts of DNA duplexes or in non-duplex DNA structures. ***NtC*** *AB1S*, *BBS1*, *BB2S*, and *NS1S* occur in guanine tetraplexes, *BB1S* and *BB2S* are actually observed only in their G-G steps 1jpq_A_DT1008_DG1009 ((Haider *et al.*, 2002)). The structural similarity of *NS1S* and *NS02* explains their similar structural role: both form a bridge between the loop and the G-quartet of a tetraplex. *NS1S* is found almost exclusively entering the G-quartet by the TG sequence (1jpq_A_DT1008_DG1009), and *NS02* leaving it by GT (4fxm_A_DG10_DT11 (Nicoludis *et al.*, 2012)). *NS02* can, however, be found in other than tetraplex structures such as a hairpin loop (3c2p_D_DA16_DA17 (Gleghorn *et al.*, 2008)), or in a topologically complex self-folding loop in 4f4y_P_DG13_DC14 (Wilson *et al.*, 2013). *BBS1* also forms the G-quartet core of tetraplex structures (1jpq_A_DG1003_DG1004), but its structural role is much wider. It accommodates a purine-purine mismatch (178d_B_8OG21_DG22 (McAuley-Hecht *et al.*, 1994)), occurs in loops of complicated self-folding ssDNA hairpin structures (2vju_C_DA-20_DG-19 (Barabas *et al.*, 2008)), or in an unusual duplex linking Z-DNA ends with T-T mismatches in the duplex midpoint (4l25_B_DG4_DA5 (Kondo *et al.*, 2014)). *BBS1*, together with another unusual conformer, *AB1S*, is involved in forming non-Watson-Crick C-G pairs in TATA box DNA bound to the TBP protein (e.g. 1qn7_E_DG210_DG211, 1qn3_E_DC209_DG210 (Patikoglou *et al.*, 1999)). The DNA duplex resembles A-form in

these complexes but, as can be shown by a detailed conformational analysis at dnatco.org, it is severely deformed at several places. Suboptimal hydrogen bonding by the guanine Hoogsteen edge to the cytosine C-H edge then probably releases strain of the deformed duplex. *AB1S* may accommodate steric strain in duplexes caused by mismatched pairs such as G-G mismatch in 1nk4 (1nk4_B_DG28_DG29 (Johnson & Beese, 2004)), or at strands opposing an abasic site of DNA lesions (3cvv (Glas *et al.*, 2009)). The last conformer belonging to the *SQX* letter, *NS05*, displays RNA-like "open" conformation with completely unstacked bases. However, the general validity of this conformer should be taken with caution as all known examples are TC steps originating from DNA complexes with DNA polymerase rb69 (3nci_T_DT1_DC2 (Wang *et al.*, 2011)).

*ZZZ: Z-form conformers*. The four conformers building the left handed Z-DNA duplex are so called ZI (*ZZS1*) and ZII (*ZZS2*) forms of the purine-pyrimidine steps. The alternating pyrimidine-purine steps are also described by two conformers, the major *ZZ1S*, and the minor *ZZS2* with δ1 in the C2'-*endo* pucker, which can occur only at the strand ends. In all *ZZZ* conformers, a base in the *syn* orientation is always purine.

*NAN: non-assigned conformers*. About 18 % of the analyzed steps end up as unassigned to any of the existing ***NtC***. Although the assignment process can be tuned to classify a higher number of dinucleotides by loosening parameters of the currently used protocol, we consider it representing a realistic compromise between the assignment accuracy and the complex nature of the DNA conformational space.

# *Annotation of selected prototypical DNA structures.*

**Table S3**. The assignment of the ***NtC*** classes to selected crystal, NMR, and MD dodecamer structures. a) ***NtC*** classes of structures appearing in Figure 6 in the main text. The assignment is also available at the web site dnatco.org.

***CANA*** *assignment for structures shown in Figure 6*

| Conner, B.N: JMB 174: 663-695, '84 | | Drew, H.R.: PNAS 78: 2179-2183, '81 | | Harper, A.: Acta D 54: 1273-1284, '98 | | Chen, L.: BCH 33: 13540-13546, '94 | |
|---|---|---|---|---|---|---|---|
| step | ntC | step | ntC | step | ntC | step | ntC |
| 1ana_A_C381_DC2 | AA00 | 1bna_A_DC1_DG2 | BB04 | 362d_A_DT1_DG2 | ZZ1S | 190d_A_DC1_DC2 | AA02 |
| 1ana_A_DC2_DG3 | AA00 | 1bna_A_DG2_DC3 | BA05 | 362d_A_DG2_DC3 | ZZS2 | 190d_A_DC2_DC3 | NANT |
| 1ana_A_DG3_DG4 | AB01 | 1bna_A_DC3_DG4 | AB01 | 362d_A_DC3_DG4 | ZZ1S | 190d_A_DC3_DC4 | NANT |
| 1ana_B_C385_DC6 | AA00 | 1bna_A_DG4_DA5 | BB04 | 362d_A_DG4_DC5 | ZZS1 | 190d_B_DC5_DC6 | NANT |
| 1ana_B_DC6_DG7 | AA00 | 1bna_A_DA5_DA6 | BB01 | 362d_A_DC5_DA6 | ZZ1S | 190d_B_DC6_DC7 | NANT |
| 1ana_B_DG7_DG8 | AA00 | 1bna_A_DA6_DT7 | BA05 | 362d_B_DT7_DG8 | ZZ1S | 190d_B_DC7_DC8 | NANT |
| | | 1bna_A_DT7_DT8 | BA05 | 362d_B_DG8_DC9 | ZZS2 | 190d_C_DC9_DC10 | NANT |
| | | 1bna_A_DT8_DC9 | BB01 | 362d_B_DC9_DG10 | ZZ1S | 190d_C_DC10_DC11 | AA02 |
| | | 1bna_A_DC9_DG10 | BB00 | 362d_B_DG10_DC11 | ZZS1 | 190d_C_DC11_DC12 | NANT |
| | | 1bna_A_DG10_DC11 | BB07 | 362d_B_DC11_DA12 | ZZ2S | 190d_D_DC13_DC14 | AA02 |
| | | 1bna_A_DC11_DG12 | BA05 | | | 190d_D_DC14_DC15 | AA02 |
| | | 1bna_B_DC13_DG14 | BB00 | | | 190d_D_DC15_DC16 | AB01 |
| | | 1bna_B_DG14_DC15 | BA05 | | | | |
| | | 1bna_B_DC15_DG16 | AB01 | | | | |
| | | 1bna_B_DG16_DA17 | BB00 | | | | |
| | | 1bna_B_DA17_DA18 | BB00 | | | | |
| | | 1bna_B_DA18_DT19 | BA05 | | | | |
| | | 1bna_B_DT19_DT20 | BB01 | | | | |
| | | 1bna_B_DT20_DC21 | BB01 | | | | |
| | | 1bna_B_DC21_DG22 | BB00 | | | | |
| | | 1bna_B_DG22_DC23 | BA17 | | | | |
| | | 1bna_B_DC23_DG24 | BA05 | | | | |

| Haider, S.: JMB 320: 189-200, '02 | | Eichman, B.F.: PNAS 97: 3971-3976, '00 | | | |
|---|---|---|---|---|---|
| step | ntC | step | ntC | step | ntC |
| 1jpq_A_DG1001_DG1002 | BBS1 | 1dcw_A_DC1_DC2 | BB16 | 1dcw_C_DC1_DC2 | BB16 |
| 1jpq_A_DG1002_DG1003 | BB2S | 1dcw_A_DC2_DG3 | BB07 | 1dcw_C_DC2_DG3 | BB07 |
| 1jpq_A_DG1003_DG1004 | BBS1 | 1dcw_A_DG3_DG4 | BB07 | 1dcw_C_DG3_DG4 | BB07 |
| 1jpq_A_DG1004_BRU1005 | BA05 | 1dcw_A_DG4_DT5 | BB00 | 1dcw_C_DG4_DT5 | BB00 |
| 1jpq_A_BRU1005_DT1006 | AB01 | 1dcw_A_DT5_DA6 | NANT | 1dcw_C_DT5_DA6 | BB04 |
| 1jpq_A_DT1006_DT1007 | NS03 | 1dcw_A_DA6_DC7 | BB07 | 1dcw_C_DA6_DC7 | BB07 |
| 1jpq_A_DT1007_DT1008 | BB16 | 1dcw_A_DC7_DC8 | BB00 | 1dcw_C_DC7_DC8 | BB00 |
| 1jpq_A_DT1008_DG1009 | NS1S | 1dcw_A_DC8_DG9 | BB07 | 1dcw_C_DC8_DG9 | BB07 |
| 1jpq_A_DG1009_DG1010 | BBS1 | 1dcw_A_DG9_DG10 | BB04 | 1dcw_C_DG9_DG10 | BB04 |
| 1jpq_A_DG1010_DG1011 | BB1S | 1dcw_B_DC1_DC2 | BB16 | 1dcw_D_DC1_DC2 | BB16 |
| 1jpq_A_DG1011_DG1012 | BBS1 | 1dcw_B_DC2_DG3 | BB07 | 1dcw_D_DC2_DG3 | BB07 |
| 1jpq_B_DG2001_DG2002 | BBS1 | 1dcw_B_DG3_DG4 | BB07 | 1dcw_D_DG3_DG4 | BB07 |
| 1jpq_B_DG2002_DG2003 | BB2S | 1dcw_B_DG4_DT5 | BB00 | 1dcw_D_DG4_DT5 | BB00 |
| 1jpq_B_DG2003_DG2004 | BBS1 | 1dcw_B_DT5_DA6 | BB04 | 1dcw_D_DT5_DA6 | BB04 |
| 1jpq_B_DG2004_BRU2005 | BA05 | 1dcw_B_DA6_DC7 | NS04 | 1dcw_D_DA6_DC7 | NS04 |
| 1jpq_B_BRU2005_DT2006 | AB01 | 1dcw_B_DC7_DC8 | BB16 | 1dcw_D_DC7_DC8 | BB16 |
| 1jpq_B_DT2006_DT2007 | NS03 | 1dcw_B_DC8_DG9 | BB07 | 1dcw_D_DC8_DG9 | BB07 |
| 1jpq_B_DT2007_DT2008 | BB16 | 1dcw_B_DG9_DG10 | BB07 | 1dcw_D_DG9_DG10 | BB07 |
| 1jpq_B_DT2008_DG2009 | NS1S | | | | |
| 1jpq_B_DG2009_DG2010 | NANT | | | | |
| 1jpq_B_DG2010_DG2011 | BB1S | | | | |
| 1jpq_B_DG2011_DG2012 | BBS1 | | | | |

**Table S3**. The assignment of the ***NtC*** classes to selected DNA structures. b) ***NtC*** classes of crystal, NMR, and MD structures of oligonucleotides with sequence of the Dickerson-Drew dodecamer. The assignment is also available at the web site dnatco.org.

*Selected X-ray structures*

| Drew, H.R., PNAS 78: 2179-2183, '81 | | Drew, H.R., PNAS 79: 4040-4044, '82 | | Holbrook, S.R., Acta B 41: 255-262, '85 | | Westhof, E., JBSD 5: 581-600, '87 | |
|---|---|---|---|---|---|---|---|
| step | Ddnc | step | Ddnc | step | Ddnc | step | Ddnc |
| 1bna_A_DC1_DG2 | BB04 | 2bna_A_DC1_DG2 | BB04 | 7bna_A_DC1_DG2 | NANT | 9bna_A_DC1_DG2 | BB01 |
| 1bna_A_DG2_DC3 | BA05 | 2bna_A_DG2_DC3 | BA01 | 7bna_A_DG2_DC3 | BA05 | 9bna_A_DG2_DC3 | BA01 |
| 1bna_A_DC3_DG4 | AB01 | 2bna_A_DC3_DG4 | AB01 | 7bna_A_DC3_DG4 | AB01 | 9bna_A_DC3_DG4 | AB01 |
| 1bna_A_DG4_DA5 | BB04 | 2bna_A_DG4_DA5 | BB04 | 7bna_A_DG4_DA5 | BB04 | 9bna_A_DG4_DA5 | BB00 |
| 1bna_A_DA5_DA6 | BB01 | 2bna_A_DA5_DA6 | BB00 | 7bna_A_DA5_DA6 | BB01 | 9bna_A_DA5_DA6 | BB01 |
| 1bna_A_DA6_DT7 | BA05 | 2bna_A_DA6_DT7 | BA05 | 7bna_A_DA6_DT7 | BB01 | 9bna_A_DA6_DT7 | BA05 |
| 1bna_A_DT7_DT8 | BA05 | 2bna_A_DT7_DT8 | BB01 | 7bna_A_DT7_DT8 | BB01 | 9bna_A_DT7_DT8 | BB01 |
| 1bna_A_DT8_DC9 | BB01 | 2bna_A_DT8_DC9 | BB01 | 7bna_A_DT8_DC9 | BB00 | 9bna_A_DT8_DC9 | BB00 |
| 1bna_A_DC9_DG10 | BB00 | 2bna_A_DC9_DG10 | BB04 | 7bna_A_DC9_DG10 | BB15 | 9bna_A_DC9_DG10 | BB00 |
| 1bna_A_DG10_DC11 | BB07 | 2bna_A_DG10_DC11 | BB07 | 7bna_A_DG10_DC11 | BB07 | 9bna_A_DG10_DC11 | NANT |
| 1bna_A_DC11_DG12 | BA05 | 2bna_A_DC11_DG12 | BA05 | 7bna_A_DC11_DG12 | BA05 | 9bna_A_DC11_DG12 | BB00 |
| 1bna_B_DC13_DG14 | BB00 | 2bna_B_DC13_DG14 | BB04 | 7bna_B_DC13_DG14 | BB00 | 9bna_B_DC13_DG14 | BB00 |
| 1bna_B_DG14_DC15 | BA05 | 2bna_B_DG14_DC15 | BA05 | 7bna_B_DG14_DC15 | BA05 | 9bna_B_DG14_DC15 | BA05 |
| 1bna_B_DC15_DG16 | AB01 | 2bna_B_DC15_DG16 | AB01 | 7bna_B_DC15_DG16 | AB01 | 9bna_B_DC15_DG16 | AB01 |
| 1bna_B_DG16_DA17 | BB00 | 2bna_B_DG16_DA17 | BB00 | 7bna_B_DG16_DA17 | BB00 | 9bna_B_DG16_DA17 | BB00 |
| 1bna_B_DA17_DA18 | BB00 | 2bna_B_DA17_DA18 | BB00 | 7bna_B_DA17_DA18 | BB00 | 9bna_B_DA17_DA18 | BA05 |
| 1bna_B_DA18_DT19 | BA05 | 2bna_B_DA18_DT19 | BB01 | 7bna_B_DA18_DT19 | BB01 | 9bna_B_DA18_DT19 | BA05 |
| 1bna_B_DT19_DT20 | BB01 | 2bna_B_DT19_DT20 | BB00 | 7bna_B_DT19_DT20 | BB01 | 9bna_B_DT19_DT20 | BB01 |
| 1bna_B_DT20_DC21 | BB01 | 2bna_B_DT20_DC21 | BB01 | 7bna_B_DT20_DC21 | BB01 | 9bna_B_DT20_DC21 | BB01 |
| 1bna_B_DC21_DG22 | BB00 | 2bna_B_DC21_DG22 | BB00 | 7bna_B_DC21_DG22 | BB00 | 9bna_B_DC21_DG22 | BB00 |
| 1bna_B_DG22_DC23 | BA17 | 2bna_B_DG22_DC23 | BA17 | 7bna_B_DG22_DC23 | BB07 | 9bna_B_DG22_DC23 | BB07 |
| 1bna_B_DC23_DG24 | BA05 | 2bna_B_DC23_DG24 | AA00 | 7bna_B_DC23_DG24 | BA01 | 9bna_B_DC23_DG24 | BB01 |

| Aymami, J., PNAS 87: 2526-2530, '90 | | Aymami, J., PNAS 87: 2526-2530, '90 | | Chiu, T.K., JMB 301: 915-945, '00 | | Chiu, T.K., JMB 301: 915-945, '00 | |
|---|---|---|---|---|---|---|---|
| step | Ddnc | step | Ddnc | step | Ddnc | step | Ddnc |
| 1vte_A_DC1_DG2 | NANT | 1ndn_A_DC1_DG2 | NANT | 1en3_A_DC1_DC2 | BB00 | 1en8_A_DC1_DC2 | AB01 |
| 1vte_A_DG2_DC3 | NANT | 1ndn_A_DG2_DC3 | NANT | 1en3_A_DC2_DA3 | BB07 | 1en8_A_DC2_DA3 | BB07 |
| 1vte_A_DC3_DG4 | NANT | 1ndn_A_DC3_DG4 | BB10 | 1en3_A_DA3_DA4 | BA05 | 1en8_A_DA3_DA4 | BB00 |
| 1vte_A_DG4_DA5 | NANT | 1ndn_A_DG4_DA5 | BB10 | 1en3_A_DA4_DC5 | BB01 | 1en8_A_DA4_DC5 | BB00 |
| 1vte_A_DA5_DA6 | NANT | 1ndn_A_DA5_DA6 | NANT | 1en3_A_DC5_DG6 | BB00 | 1en8_A_DC5_DG6 | BB07 |
| 1vte_A_DA6_DA7 | NANT | 1ndn_A_DA6_DA7 | NANT | 1en3_A_DG6_DT7 | BB01 | 1en8_A_DG6_DT7 | BB01 |
| 1vte_A_DA7_DA8 | BB00 | 1ndn_A_DA7_DA8 | NANT | 1en3_A_DT7_DT8 | BB01 | 1en8_A_DT7_DT8 | BB00 |
| 1vte_A_DA8_DC9 | BB02 | 1ndn_A_DA8_DC9 | NANT | 1en3_A_DT8_DG9 | BB07 | 1en8_A_DT8_DG9 | BB07 |
| 1vte_A_DC9_DG10 | NANT | 1ndn_A_DC9_DG10 | NANT | 1en3_A_DG9_DG10 | BB00 | 1en8_A_DG9_DG10 | BA05 |
| 1vte_A_DG10_DC11 | AB01 | 1ndn_A_DG10_DC11 | NANT | | | | |
| 1vte_A_DC11_DG12 | NANT | 1ndn_A_DC11_DG12 | AA02 | | | | |
| 1vte_B_DC13_DG14 | NANT | 1ndn_B_DC13_DG14 | NANT | 1en3_A_DC1_DC2 | BB00 | 1en8_A_DC1_DC2 | AB01 |
| 1vte_B_DG14_DC15 | NANT | 1ndn_B_DG14_DC15 | NANT | 1en3_A_DC2_DA3 | BB07 | 1en8_A_DC2_DA3 | BB07 |
| 1vte_B_DC15_DG16 | NANT | 1ndn_B_DC15_DG16 | NANT | 1en3_A_DA3_DA4 | BA05 | 1en8_A_DA3_DA4 | BB00 |
| 1vte_B_DG16_DT17 | NANT | 1ndn_B_DG16_DT17 | NANT | 1en3_A_DA4_DC5 | BB01 | 1en8_A_DA4_DC5 | BB00 |
| 1vte_B_DT17_DT18 | NANT | 1ndn_B_DT17_DT18 | NANT | 1en3_A_DC5_DG6 | BB00 | 1en8_A_DC5_DG6 | BB07 |
| | | | | 1en3_A_DG6_DT7 | BB01 | 1en8_A_DG6_DT7 | BB01 |
| 1vte_C_DT19_DT20 | NANT | 1ndn_C_DT19_DT20 | NANT | 1en3_A_DT7_DT8 | BB01 | 1en8_A_DT7_DT8 | BB00 |
| 1vte_C_DT20_DC21 | AA01 | 1ndn_C_DT20_DC21 | BB02 | 1en3_A_DT8_DG9 | BB07 | 1en8_A_DT8_DG9 | BB07 |
| 1vte_C_DC21_DG22 | NANT | 1ndn_C_DC21_DG22 | NANT | 1en3_A_DG9_DG10 | BB00 | 1en8_A_DG9_DG10 | BA05 |
| 1vte_C_DG22_DC23 | NANT | 1ndn_C_DG22_DC23 | NANT | | | | |
| 1vte_C_DC23_DG24 | NANT | 1ndn_C_DC23_DG24 | NANT | | | | |

| Chiu, T.K.,JMB 301: 915-945, '00 | | Chiu, T.K.,JMB 301: 915-945, '00 | | Sines, C.C., JACS 122: 11048, '00 | | Liu, J., JBC 274: 24749-24752, '99 | |
|---|---|---|---|---|---|---|---|
| step | Ddnc | step | Ddnc | step | Ddnc | step | Ddnc |
| 1en9_A_DC1_DC2 | BB00 | 1ene_A_DC1_DC2 | BB00 | 1fq2_A_DC1_DG2 | BB00 | | |
| 1en9_A_DC2_DA3 | BB07 | 1ene_A_DC2_DA3 | BB07 | 1fq2_A_DG2_DC3 | NANT | 463d_A_DG2_DC3 | BA01 |
| 1en9_A_DA3_DG4 | BB00 | 1ene_A_DA3_DG4 | BB00 | 1fq2_A_DC3_DG4 | AB01 | 463d_A_DC3_DG4 | AB01 |
| 1en9_A_DG4_DC5 | BB07 | 1ene_A_DG4_DC5 | BB07 | 1fq2_A_DG4_DA5 | BB00 | 463d_A_DG4_DA5 | BB00 |
| 1en9_A_DC5_DG6 | BB00 | 1ene_A_DC5_DG6 | BB00 | 1fq2_A_DA5_DA6 | BB01 | 463d_A_DA5_DA6 | BB00 |
| 1en9_A_DG6_DC7 | BA05 | 1ene_A_DG6_DC7 | BA05 | 1fq2_A_DA6_DT7 | BA05 | 463d_A_DA6_DT7 | BB01 |
| 1en9_A_DC7_DT8 | AB01 | 1ene_A_DC7_DT8 | AB01 | 1fq2_A_DT7_DT8 | BB01 | 463d_A_DT7_DT8 | BA05 |
| 1en9_A_DT8_DG9 | BB07 | 1ene_A_DT8_DG9 | BB07 | 1fq2_A_DT8_DC9 | BB01 | 463d_A_DT8_DC9 | BB01 |
| 1en9_A_DG9_DG10 | BB00 | 1ene_A_DG9_DG10 | BB00 | 1fq2_A_DC9_DG10 | BB00 | 463d_A_DC9_DG10 | NANT |
| | | | | 1fq2_A_DG10_DC11 | BB07 | 463d_A_DG10_DC11 | BB07 |
| | | | | 1fq2_A_DC11_DG12 | NANT | 463d_A_DC11_DG12 | NANT |
| 1en9_A_DC1_DC2 | BB00 | 1ene_A_DC1_DC2 | BB00 | 1fq2_B_DC1_DG2 | BA05 | | |
| 1en9_A_DC2_DA3 | BB07 | 1ene_A_DC2_DA3 | BB07 | 1fq2_B_DG2_DC3 | BA05 | 463d_B_DG14_DC15 | BB00 |
| 1en9_A_DA3_DG4 | BB00 | 1ene_A_DA3_DG4 | BB00 | 1fq2_B_DC3_DG4 | AB01 | 463d_B_DC15_DG16 | BB00 |
| 1en9_A_DG4_DC5 | BB07 | 1ene_A_DG4_DC5 | BB07 | 1fq2_B_DG4_DA5 | BB00 | 463d_B_DG16_DA17 | BB00 |
| 1en9_A_DC5_DG6 | BB00 | 1ene_A_DC5_DG6 | BB00 | 1fq2_B_DA5_DA6 | BA05 | 463d_B_DA17_DA18 | BA05 |
| 1en9_A_DG6_DC7 | BA05 | 1ene_A_DG6_DC7 | BA05 | 1fq2_B_DA6_DT7 | BB01 | 463d_B_DA18_DT19 | BB01 |
| 1en9_A_DC7_DT8 | AB01 | 1ene_A_DC7_DT8 | AB01 | 1fq2_B_DT7_DT8 | BB01 | 463d_B_DT19_DT20 | BB01 |
| 1en9_A_DT8_DG9 | BB07 | 1ene_A_DT8_DG9 | BB07 | 1fq2_B_DT8_DC9 | BA05 | 463d_B_DT20_DC21 | BA05 |
| 1en9_A_DG9_DG10 | BB00 | 1ene_A_DG9_DG10 | BB00 | 1fq2_B_DC9_DG10 | AB01 | 463d_B_DC21_DG22 | AB01 |
| | | | | 1fq2_B_DG10_DC11 | BA08 | 463d_B_DG22_DC23 | BB07 |
| | | | | 1fq2_B_DC11_DG12 | AA00 | 463d_B_DC23_DG24 | NANT |

| Tsunoda, M., Acta D 57: 345-348, '01 | | Tsunoda, M., Acta D 57: 345-348, '01 | | Tsunoda, M., Acta D 57: 345-348, '01 | | Tsunoda, M., Acta D 57: 345-348, '01 | |
|---|---|---|---|---|---|---|---|
| step | Ddnc | step | Ddnc | step | Ddnc | step | Ddnc |
| 1g75_A_DC1_DG2 | BB00 | 1g8n_A_DC1_DG2 | BB00 | 1g8u_A_DC1_DG2 | BA10 | 1g8v_A_DC1_DG2 | BB04 |
| 1g75_A_DG2_DC3 | BA01 | 1g8n_A_DG2_DC3 | BA01 | 1g8u_A_DG2_DC3 | AA00 | 1g8v_A_DG2_DC3 | BA05 |
| 1g75_A_DC3_DG4 | AB01 | 1g8n_A_DC3_DG4 | AB01 | 1g8u_A_DC3_DG4 | AB01 | 1g8v_A_DC3_DG4 | AB01 |
| 1g75_A_DG4_DA5 | BB00 | 1g8n_A_DG4_DA5 | BB01 | 1g8u_A_DG4_DA5 | BB01 | 1g8v_A_DG4_DA5 | BB01 |
| 1g75_A_DA5_DA6 | BB01 | 1g8n_A_DA5_DA6 | BB01 | 1g8u_A_DA5_DA6 | BB01 | 1g8v_A_DA5_DA6 | BB00 |
| 1g75_A_DA6_DT7 | BA05 | 1g8n_A_DA6_DT7 | BA05 | 1g8u_A_DA6_DT7 | BB01 | 1g8v_A_DA6_DT7 | BB01 |
| | | | | | | | |
| 1g75_A_DC9_DG10 | BB00 | 1g8n_A_DC9_DG10 | BB00 | 1g8u_A_DC9_DG10 | BB00 | 1g8v_A_DC9_DG10 | BB00 |
| 1g75_A_DG10_DC11 | BB07 | 1g8n_A_DG10_DC11 | BB07 | 1g8u_A_DG10_DC11 | BB07 | 1g8v_A_DG10_DC11 | BB07 |
| 1g75_A_DC11_DG12 | BA05 | 1g8n_A_DC11_DG12 | BA05 | 1g8u_A_DC11_DG12 | BA05 | 1g8v_A_DC11_DG12 | BA05 |
| 1g75_B_DC13_DG14 | BB00 | 1g8n_B_DC13_DG14 | BA05 | 1g8u_B_DC13_DG14 | BB04 | 1g8v_B_DC13_DG14 | BB07 |
| 1g75_B_DG14_DC15 | BA05 | 1g8n_B_DG14_DC15 | BA05 | 1g8u_B_DG14_DC15 | BA05 | 1g8v_B_DG14_DC15 | BA01 |
| 1g75_B_DC15_DG16 | AB01 | 1g8n_B_DC15_DG16 | AB01 | 1g8u_B_DC15_DG16 | AB01 | 1g8v_B_DC15_DG16 | AB01 |
| 1g75_B_DG16_DA17 | BB00 | 1g8n_B_DG16_DA17 | BB00 | 1g8u_B_DG16_DA17 | BB00 | 1g8v_B_DG16_DA17 | BB00 |
| 1g75_B_DA17_DA18 | BB01 | 1g8n_B_DA17_DA18 | BB01 | 1g8u_B_DA17_DA18 | BB01 | 1g8v_B_DA17_DA18 | BB01 |
| 1g75_B_DA18_DT19 | BA05 | 1g8n_B_DA18_DT19 | BA05 | 1g8u_B_DA18_DT19 | BB01 | 1g8v_B_DA18_DT19 | BB01 |
| | | | | | | | |
| 1g75_B_DC21_DG22 | BB00 | 1g8n_B_DC21_DG22 | BB00 | 1g8u_B_DC21_DG22 | BB00 | 1g8v_B_DC21_DG22 | BB00 |
| 1g75_B_DG22_DC23 | BA08 | 1g8n_B_DG22_DC23 | BA08 | 1g8u_B_DG22_DC23 | BA17 | 1g8v_B_DG22_DC23 | BA08 |
| 1g75_B_DC23_DG24 | AA00 | 1g8n_B_DC23_DG24 | AA00 | 1g8u_B_DC23_DG24 | BA05 | 1g8v_B_DC23_DG24 | AA00 |

| Wilds, C.J., JACS 124: 13716-13721, '02 | | Moulaei, T., BCH 44: 7458-7468, '05 | | Fratini, A.V., JBC 257: 14686-14707, '82 | | Chiu, T.K., JMB 292: 589-608, '99 | |
|---|---|---|---|---|---|---|---|
| step | Ddnc | step | Ddnc | step | Ddnc | step | Ddnc |
| 1n1o_A_DC1_DG2 | BB00 | 1z5t_A_DC1_DG2 | BB04 | 3bna_A_DC1_DG2 | NANT | 455d_A_DC1_DG2 | BB00 |
| 1n1o_A_DG2_DC3 | BA01 | 1z5t_A_DG2_DC3 | BA01 | 3bna_A_DG2_DC3 | BA05 | 455d_A_DG2_DC3 | BA01 |
| 1n1o_A_DC3_DG4 | AB01 | 1z5t_A_DC3_DG4 | AB01 | 3bna_A_DC3_DG4 | BB16 | 455d_A_DC3_DG4 | AB01 |
| 1n1o_A_DG4_DA5 | BB00 | 1z5t_A_DG4_DA5 | BB00 | 3bna_A_DG4_DA5 | BB00 | 455d_A_DG4_DA5 | BB00 |
| 1n1o_A_DA5_DA6 | BB00 | 1z5t_A_DA5_DA6 | BB00 | 3bna_A_DA5_DA6 | BB01 | 455d_A_DA5_DA6 | BB00 |
| | | | | 3bna_A_DA6_DT7 | BA05 | 455d_A_DA6_DT7 | BB01 |
| | | | | 3bna_A_DT7_DT8 | AA02 | 455d_A_DT7_DT8 | BB01 |
| 1n1o_A_DT8_DC9 | BB00 | | | 3bna_A_DT8_CBR9 | BA05 | 455d_A_DT8_DC9 | BB00 |
| 1n1o_A_DC9_DG10 | BB00 | 1z5t_A_DC9_DG10 | BB00 | 3bna_A_CBR9_DG10 | AB01 | 455d_A_DC9_DG10 | BB00 |
| 1n1o_A_DG10_DC11 | BB07 | 1z5t_A_DG10_DC11 | BB07 | 3bna_A_DG10_DC11 | BB07 | 455d_A_DG10_DC11 | BB07 |
| 1n1o_A_DC11_DG12 | BB00 | 1z5t_A_DC11_DG12 | BA05 | 3bna_A_DC11_DG12 | BA05 | 455d_A_DC11_DG12 | BA05 |
| 1n1o_B_DC113_DG114 | BB01 | 1z5t_B_DC13_DG14 | BB07 | 3bna_B_DC13_DG14 | BB04 | 455d_B_DC13_DG14 | BB07 |
| 1n1o_B_DG114_DC115 | BA05 | 1z5t_B_DG14_DC15 | BA01 | 3bna_B_DG14_DC15 | BA01 | 455d_B_DG14_DC15 | BB01 |
| 1n1o_B_DC115_DG116 | AB01 | 1z5t_B_DC15_DG16 | AB01 | 3bna_B_DC15_DG16 | AB01 | 455d_B_DC15_DG16 | NANT |
| 1n1o_B_DG116_DA117 | BB00 | 1z5t_B_DG16_DA17 | BB00 | 3bna_B_DG16_DA17 | BB00 | 455d_B_DG16_DA17 | BB00 |
| 1n1o_B_DA117_DA118 | BB00 | 1z5t_B_DA17_DA18 | BB01 | 3bna_B_DA17_DA18 | BA05 | 455d_B_DA17_DA18 | BB01 |
| | | | | 3bna_B_DA18_DT19 | AA00 | 455d_B_DA18_DT19 | BB01 |
| | | | | 3bna_B_DT19_DT20 | AA02 | 455d_B_DT19_DT20 | BB01 |
| 1n1o_B_DT120_DC121 | BA05 | | | 3bna_B_DT20_CBR21 | BA05 | 455d_B_DT20_DC21 | BB00 |
| 1n1o_B_DC121_DG122 | AB01 | 1z5t_B_DC21_DG22 | BB00 | 3bna_B_CBR21_DG22 | AB01 | 455d_B_DC21_DG22 | BB00 |
| 1n1o_B_DG122_DC123 | BA08 | 1z5t_B_DG22_DC23 | BA17 | 3bna_B_DG22_DC23 | BB07 | 455d_B_DG22_DC23 | NANT |
| 1n1o_B_DC123_DG124 | AA00 | 1z5t_B_DC23_DG24 | AA02 | 3bna_B_DC23_DG24 | BA01 | 455d_B_DC23_DG24 | BA05 |

| Kowal, E.A., NAR 41: 7566-7576, '13 | | Szulik, M.W., BCH 54: 1294-1305, '15 | | Jiang, S., TBP | | Theruvathu, J.A., BCH 52: 8590, '13 | |
|---|---|---|---|---|---|---|---|
| step | Ddnc | step | Ddnc | step | Ddnc | step | Ddnc |
| | | 4i9v_A_DC1_DG2 | BB00 | 4kw0_A_DC1_DG2 | BB00 | 4mgw_A_DC1_DG2 | BB00 |
| 4hqi_A_DG2_DC3 | BA05 | 4i9v_A_DG2_DC3 | BA01 | 4kw0_A_DG2_DC3 | BA01 | 4mgw_A_DG4_DA5 | BB00 |
| | | 4i9v_A_DC3_DG4 | AB01 | 4kw0_A_DC3_DG4 | AB01 | 4mgw_A_DA5_DA6 | BB00 |
| | | 4i9v_A_DG4_DA5 | BB07 | 4kw0_A_DG4_DA5 | BB00 | 4mgw_A_DA6_DT7 | BB01 |
| 4hqi_A_DA5_DA6 | BB01 | 4i9v_A_DA5_DA6 | BB01 | 4kw0_A_DA5_DA6 | BB01 | 4mgw_A_DT7_DT8 | BB01 |
| 4hqi_A_DA6_DT7 | BA05 | 4i9v_A_DA6_DT7 | BA05 | 4kw0_A_DA6_DT7 | BA05 | 4mgw_A_DT8_DC9 | BB00 |
| 4hqi_A_DT7_DT8 | BB01 | 4i9v_A_DT7_DT8 | BB01 | 4kw0_A_DT7_DT8 | BB01 | 4mgw_A_DC9_DG10 | BB00 |
| | | | | 4kw0_A_DT8_DC9 | BB00 | | |
| 4hqi_A_DG10_DC11 | BB04 | 4i9v_A_DG10_DC11 | BB07 | | | 4mgw_A_DG10_DC11 | BB07 |
| 4hqi_A_DC11_DG12 | NANT | 4i9v_A_DC11_DG12 | BB00 | 4kw0_A_DC11_DG12 | BB00 | 4mgw_A_DC11_DG12 | BA05 |
| | | 4i9v_B_DC13_DG14 | BA05 | 4kw0_B_DC13_DG14 | BB01 | 4mgw_B_DC13_DG14 | BB07 |
| 4hqi_B_DG14_DC15 | BB01 | 4i9v_B_DG14_DC15 | BA05 | 4kw0_B_DG14_DC15 | BA05 | | |
| | | 4i9v_B_DC15_DG16 | AB01 | 4kw0_B_DC15_DG16 | AB01 | | |
| | | 4i9v_B_DG16_DA17 | BB00 | 4kw0_B_DG16_DA17 | BB00 | 4mgw_B_DG16_DA17 | BB00 |
| 4hqi_B_DA17_DA18 | BB07 | 4i9v_B_DA17_DA18 | BB01 | 4kw0_B_DA17_DA18 | BB01 | 4mgw_B_DA17_DA18 | BB00 |
| 4hqi_B_DA18_DT19 | BA05 | 4i9v_B_DA18_DT19 | BA05 | 4kw0_B_DA18_DT19 | BB01 | 4mgw_B_DA18_DT19 | BB01 |
| 4hqi_B_DT19_DT20 | AB01 | 4i9v_B_DT19_DT20 | BB01 | 4kw0_B_DT19_DT20 | BB01 | 4mgw_B_DT19_DT20 | BB01 |
| | | | | 4kw0_B_DT20_DC21 | BB01 | 4mgw_B_DT20_DC21 | BB00 |
| | | | | | | 4mgw_B_DC21_DG22 | BB00 |
| 4hqi_B_DG22_DC23 | BB04 | 4i9v_B_DG22_DC23 | BA08 | | | 4mgw_B_DG22_DC23 | BA08 |
| 4hqi_B_DC23_DG24 | NANT | 4i9v_B_DC23_DG24 | AA00 | 4kw0_B_DC23_DG24 | BA01 | 4mgw_B_DC23_DG24 | AA00 |

## *X-ray structures with drugs bound in the minor groove*

| Wood, A.A., NAR 23: 3678-3684, '95 | | Quintana, J.R., BCH 30: 10294, '91 | | Quintana, J.R., BCH 30: 10294, '91 | | Quintana, J.R., BCH 30: 10294, '91 | |
|---|---|---|---|---|---|---|---|
| step | Ddnc | step | Ddnc | step | Ddnc | step | Ddnc |
| 109d_A_DC1_DG2 | BB00 | 1d43_A_DC1_DG2 | BB00 | 1d44_A_DC1_DG2 | BB00 | 1d45_A_DC1_DG2 | BB00 |
| 109d_A_DG2_DC3 | BB00 | 1d43_A_DG2_DC3 | BA01 | 1d44_A_DG2_DC3 | BA01 | 1d45_A_DG2_DC3 | BA01 |
| 109d_A_DC3_DG4 | BB04 | 1d43_A_DC3_DG4 | AB01 | 1d44_A_DC3_DG4 | AB01 | 1d45_A_DC3_DG4 | AB01 |
| 109d_A_DG4_DA5 | BB04 | 1d43_A_DG4_DA5 | BB04 | 1d44_A_DG4_DA5 | BB00 | 1d45_A_DG4_DA5 | BB00 |
| 109d_A_DA5_DA6 | BB00 | 1d43_A_DA5_DA6 | BB00 | 1d44_A_DA5_DA6 | BB01 | 1d45_A_DA5_DA6 | BB00 |
| 109d_A_DA6_DT7 | BB01 | 1d43_A_DA6_DT7 | BA05 | 1d44_A_DA6_DT7 | BA05 | 1d45_A_DA6_DT7 | BA05 |
| 109d_A_DT7_DT8 | BB00 | 1d43_A_DT7_DT8 | NANT | 1d44_A_DT7_DT8 | NANT | 1d45_A_DT7_DT8 | NANT |
| 109d_A_DT8_DC9 | BB00 | 1d43_A_DT8_DC9 | NANT | 1d44_A_DT8_DC9 | NANT | 1d45_A_DT8_DC9 | NANT |
| 109d_A_DC9_DG10 | BB00 | 1d43_A_DC9_DG10 | BB04 | 1d44_A_DC9_DG10 | BB04 | 1d45_A_DC9_DG10 | NANT |
| 109d_A_DG10_DC11 | BB07 | 1d43_A_DG10_DC11 | BB07 | 1d44_A_DG10_DC11 | BB07 | 1d45_A_DG10_DC11 | NANT |
| 109d_A_DC11_DG12 | BB00 | 1d43_A_DC11_DG12 | NANT | 1d44_A_DC11_DG12 | NANT | 1d45_A_DC11_DG12 | BB00 |
| 109d_B_DC13_DG14 | BB07 | 1d43_B_DC13_DG14 | NANT | 1d44_B_DC13_DG14 | NANT | 1d45_B_DC13_DG14 | BB07 |
| 109d_B_DG14_DC15 | BB00 | 1d43_B_DG14_DC15 | BA05 | 1d44_B_DG14_DC15 | AA02 | 1d45_B_DG14_DC15 | BA05 |
| 109d_B_DC15_DG16 | BB04 | 1d43_B_DC15_DG16 | AB01 | 1d44_B_DC15_DG16 | AB01 | 1d45_B_DC15_DG16 | AB01 |
| 109d_B_DG16_DA17 | BB00 | 1d43_B_DG16_DA17 | NANT | 1d44_B_DG16_DA17 | NANT | 1d45_B_DG16_DA17 | BB04 |
| 109d_B_DA17_DA18 | BB00 | 1d43_B_DA17_DA18 | BB04 | 1d44_B_DA17_DA18 | BB04 | 1d45_B_DA17_DA18 | BB00 |
| 109d_B_DA18_DT19 | BB00 | 1d43_B_DA18_DT19 | BA05 | 1d44_B_DA18_DT19 | BB00 | 1d45_B_DA18_DT19 | BA01 |
| 109d_B_DT19_DT20 | BB00 | 1d43_B_DT19_DT20 | BB01 | 1d44_B_DT19_DT20 | BB01 | 1d45_B_DT19_DT20 | AA02 |
| 109d_B_DT20_DC21 | BB01 | 1d43_B_DT20_DC21 | BB01 | 1d44_B_DT20_DC21 | BB01 | 1d45_B_DT20_DC21 | NANT |
| 109d_B_DC21_DG22 | BB00 | 1d43_B_DC21_DG22 | BB00 | 1d44_B_DC21_DG22 | BB00 | 1d45_B_DC21_DG22 | NANT |
| 109d_B_DG22_DC23 | BB07 | 1d43_B_DG22_DC23 | NANT | 1d44_B_DG22_DC23 | NANT | 1d45_B_DG22_DC23 | BA17 |
| 109d_B_DC23_DG24 | BB02 | 1d43_B_DC23_DG24 | NANT | 1d44_B_DC23_DG24 | AA03 | 1d45_B_DC23_DG24 | AA04 |

| Quintana, J.R., BCH 30: 10294, '91 | | Coll, M., BCH 28: 310-320, '89 | | Campbell, N.H., BMCL 16: 15-19, '06 | | Neidle, S., TBP | |
|---|---|---|---|---|---|---|---|
| step | Ddnc | step | Ddnc | step | Ddnc | step | Ddnc |
| 1d46_A_DC1_DG2 | NANT | 1dne_A_DC1_DG2 | NANT | 2b3e_A_DC1_DG2 | BB00 | 2gvr_A_DC1_DG2 | BB00 |
| 1d46_A_DG2_DC3 | NANT | 1dne_A_DG2_DC3 | NANT | 2b3e_A_DG2_DC3 | BA01 | 2gvr_A_DG2_DC3 | BA01 |
| 1d46_A_DC3_DG4 | NANT | 1dne_A_DC3_DG4 | NANT | 2b3e_A_DC3_DG4 | AB01 | 2gvr_A_DC3_DG4 | AB01 |
| 1d46_A_DG4_DA5 | BB07 | 1dne_A_DG4_DA5 | NANT | 2b3e_A_DG4_DA5 | BB00 | 2gvr_A_DG4_DA5 | BB00 |
| 1d46_A_DA5_DA6 | BA05 | 1dne_A_DA5_DT6 | NANT | 2b3e_A_DA5_DA6 | BB01 | 2gvr_A_DA5_DA6 | BB01 |
| 1d46_A_DA6_DT7 | NANT | 1dne_A_DT6_DA7 | NANT | 2b3e_A_DA6_DT7 | BA05 | 2gvr_A_DA6_DT7 | BB01 |
| 1d46_A_DT7_DT8 | NANT | 1dne_A_DA7_DT8 | NANT | 2b3e_A_DT7_DT8 | BB01 | 2gvr_A_DT7_DT8 | BB01 |
| 1d46_A_DT8_DC9 | NANT | 1dne_A_DT8_DC9 | NANT | 2b3e_A_DT8_DC9 | BB01 | 2gvr_A_DT8_DC9 | BB01 |
| 1d46_A_DC9_DG10 | BB04 | 1dne_A_DC9_DG10 | NANT | 2b3e_A_DC9_DG10 | BB00 | 2gvr_A_DC9_DG10 | NANT |
| 1d46_A_DG10_DC11 | BB07 | 1dne_A_DG10_DC11 | NANT | 2b3e_A_DG10_DC11 | BB07 | 2gvr_A_DG10_DC11 | BB07 |
| 1d46_A_DC11_DG12 | NANT | 1dne_A_DC11_DG12 | NANT | 2b3e_A_DC11_DG12 | BA05 | 2gvr_A_DC11_DG12 | BA05 |
| 1d46_B_DC13_DG14 | BA17 | 1dne_B_DC13_DG14 | BB13 | 2b3e_B_DC13_DG14 | BB07 | 2gvr_B_DC13_DG14 | BB07 |
| 1d46_B_DG14_DC15 | NANT | 1dne_B_DG14_DC15 | BA05 | 2b3e_B_DG14_DC15 | BA05 | 2gvr_B_DG14_DC15 | BA05 |
| 1d46_B_DC15_DG16 | AB01 | 1dne_B_DC15_DG16 | NANT | 2b3e_B_DC15_DG16 | AB01 | 2gvr_B_DC15_DG16 | AB01 |
| 1d46_B_DG16_DA17 | NANT | 1dne_B_DG16_DA17 | NANT | 2b3e_B_DG16_DA17 | BB00 | 2gvr_B_DG16_DA17 | BB00 |
| 1d46_B_DA17_DA18 | BB00 | 1dne_B_DA17_DT18 | NANT | 2b3e_B_DA17_DA18 | BB00 | 2gvr_B_DA17_DA18 | BB01 |
| 1d46_B_DA18_DT19 | NANT | 1dne_B_DT18_DA19 | NANT | 2b3e_B_DA18_DT19 | BB01 | 2gvr_B_DA18_DT19 | BB01 |
| 1d46_B_DT19_DT20 | NANT | 1dne_B_DA19_DT20 | NANT | 2b3e_B_DT19_DT20 | BB01 | 2gvr_B_DT19_DT20 | BB01 |
| 1d46_B_DT20_DC21 | BB01 | 1dne_B_DT20_DC21 | BB00 | 2b3e_B_DT20_DC21 | BA05 | 2gvr_B_DT20_DC21 | BB01 |
| 1d46_B_DC21_DG22 | BB04 | 1dne_B_DC21_DG22 | NANT | 2b3e_B_DC21_DG22 | BB00 | 2gvr_B_DC21_DG22 | BB00 |
| 1d46_B_DG22_DC23 | NANT | 1dne_B_DG22_DC23 | NANT | 2b3e_B_DG22_DC23 | BB07 | 2gvr_B_DG22_DC23 | BB07 |
| 1d46_B_DC23_DG24 | BA01 | 1dne_B_DC23_DG24 | NANT | 2b3e_B_DC23_DG24 | BA05 | 2gvr_B_DC23_DG24 | BA05 |

## *Selected NMR structures*

| Tjandra, N., JACS 122: 6190-6200, '00 | | Kuszewski, J., JACS 123: 3903-3918, '01 | | Wu, Z., JBN 26: 297-315, '03 | | Denisov, A.Y., JBSD 16: 547-568, '98 | |
|---|---|---|---|---|---|---|---|
| step | Ddnc | step | Ddnc | step | Ddnc | step | Ddnc |
| 1duf_A_DC1_DG2 | BB00 | 1gip_A_DC1_DG2 | BB00 | 1naj_A_DC1_DG2 | BB00 | 2dau_A_DC1_DG2 | BB00 |
| 1duf_A_DG2_DC3 | BB01 | 1gip_A_DG2_DC3 | BB01 | 1naj_A_DG2_DC3 | BB01 | 2dau_A_DG2_DC3 | BB00 |
| 1duf_A_DC3_DG4 | BB00 | 1gip_A_DC3_DG4 | BB00 | 1naj_A_DC3_DG4 | BB00 | 2dau_A_DC3_DG4 | BB00 |
| 1duf_A_DG4_DA5 | BB00 | 1gip_A_DG4_DA5 | BB00 | 1naj_A_DG4_DA5 | BB00 | 2dau_A_DG4_DA5 | BB00 |
| 1duf_A_DA5_DA6 | BB00 | 1gip_A_DA5_DA6 | BB00 | 1naj_A_DA5_DA6 | BB01 | 2dau_A_DA5_DA6 | BB00 |
| 1duf_A_DA6_DT7 | BB01 | 1gip_A_DA6_DT7 | BB01 | 1naj_A_DA6_DT7 | BA05 | 2dau_A_DA6_DT7 | BA05 |
| 1duf_A_DT7_DT8 | BB01 | 1gip_A_DT7_DT8 | BB01 | 1naj_A_DT7_DT8 | BB01 | 2dau_A_DT7_DT8 | BA05 |
| 1duf_A_DT8_DC9 | BA05 | 1gip_A_DT8_DC9 | BA05 | 1naj_A_DT8_DC9 | BB00 | 2dau_A_DT8_DC9 | BB01 |
| 1duf_A_DC9_DG10 | BB01 | 1gip_A_DC9_DG10 | BB00 | 1naj_A_DC9_DG10 | BB00 | 2dau_A_DC9_DG10 | BB01 |
| 1duf_A_DG10_DC11 | BA05 | 1gip_A_DG10_DC11 | BB01 | 1naj_A_DG10_DC11 | BB01 | 2dau_A_DG10_DC11 | BB00 |
| 1duf_A_DC11_DG12 | BB01 | 1gip_A_DC11_DG12 | BB01 | 1naj_A_DC11_DG12 | BB01 | 2dau_A_DC11_DG12 | BB01 |
| 1duf_B_DC13_DG14 | BB00 | 1gip_B_DC13_DG14 | BB00 | 1naj_B_DC13_DG14 | BB00 | 2dau_B_DC13_DG14 | BB00 |
| 1duf_B_DG14_DC15 | BB01 | 1gip_B_DG14_DC15 | BB01 | 1naj_B_DG14_DC15 | BB01 | 2dau_B_DG14_DC15 | BB00 |
| 1duf_B_DC15_DG16 | BB00 | 1gip_B_DC15_DG16 | BB00 | 1naj_B_DC15_DG16 | BB00 | 2dau_B_DC15_DG16 | BB00 |
| 1duf_B_DG16_DA17 | BB00 | 1gip_B_DG16_DA17 | BB00 | 1naj_B_DG16_DA17 | BB00 | 2dau_B_DG16_DA17 | BB00 |
| 1duf_B_DA17_DA18 | BB00 | 1gip_B_DA17_DA18 | BB00 | 1naj_B_DA17_DA18 | BB01 | 2dau_B_DA17_DA18 | BB00 |
| 1duf_B_DA18_DT19 | BB01 | 1gip_B_DA18_DT19 | BB01 | 1naj_B_DA18_DT19 | BA05 | 2dau_B_DA18_DT19 | BA05 |
| 1duf_B_DT19_DT20 | BB01 | 1gip_B_DT19_DT20 | BB01 | 1naj_B_DT19_DT20 | BB01 | 2dau_B_DT19_DT20 | BA05 |
| 1duf_B_DT20_DC21 | BA05 | 1gip_B_DT20_DC21 | BA05 | 1naj_B_DT20_DC21 | BB00 | 2dau_B_DT20_DC21 | BB01 |
| 1duf_B_DC21_DG22 | BB01 | 1gip_B_DC21_DG22 | BB00 | 1naj_B_DC21_DG22 | BB00 | 2dau_B_DC21_DG22 | BB01 |
| 1duf_B_DG22_DC23 | BA05 | 1gip_B_DG22_DC23 | BB01 | 1naj_B_DG22_DC23 | BB01 | 2dau_B_DG22_DC23 | BB00 |
| 1duf_B_DC23_DG24 | BB01 | 1gip_B_DC23_DG24 | BB01 | 1naj_B_DC23_DG24 | BB01 | 2dau_B_DC23_DG24 | BB01 |

## *Fiber models from AmberTools*

| AmberTools "Arnot" | | AmberTools "Langridge" | | AmberTools "Average B" | |
|---|---|---|---|---|---|
| step | Ddnc | step | Ddnc | step | Ddnc |
| arnot-fd-bdna_X_DC1_DG2 | BB00 | lang-bdna_X_DC1_DG2 | BB00 | aver-bdna_A_DC1_DG2 | NANT |
| arnot-fd-bdna_X_DG2_DC3 | BB00 | lang-bdna_X_DG2_DC3 | BB00 | aver-bdna_A_DG2_DC3 | NANT |
| arnot-fd-bdna_X_DC3_DG4 | BB00 | lang-bdna_X_DC3_DG4 | BB00 | aver-bdna_A_DC3_DG4 | NANT |
| arnot-fd-bdna_X_DG4_DA5 | BB00 | lang-bdna_X_DG4_DA5 | BB00 | aver-bdna_A_DG4_DA5 | NANT |
| arnot-fd-bdna_X_DA5_DA6 | BB00 | lang-bdna_X_DA5_DA6 | BB00 | aver-bdna_A_DA5_DA6 | BB15 |
| arnot-fd-bdna_X_DA6_DT7 | BB00 | lang-bdna_X_DA6_DT7 | BB00 | aver-bdna_A_DA6_DT7 | BB15 |
| arnot-fd-bdna_X_DT7_DT8 | BB00 | lang-bdna_X_DT7_DT8 | BB00 | aver-bdna_A_DT7_DT8 | BB15 |
| arnot-fd-bdna_X_DT8_DC9 | BB00 | lang-bdna_X_DT8_DC9 | BB00 | aver-bdna_A_DT8_DC9 | NANT |
| arnot-fd-bdna_X_DC9_DG10 | BB00 | lang-bdna_X_DC9_DG10 | BB00 | aver-bdna_A_DC9_DG10 | NANT |
| arnot-fd-bdna_X_DG10_DC11 | BB00 | lang-bdna_X_DG10_DC11 | BB00 | aver-bdna_A_DG10_DC11 | NANT |
| arnot-fd-bdna_X_DC11_DG12 | BB00 | lang-bdna_X_DC11_DG12 | BB00 | aver-bdna_A_DC11_DG12 | NANT |
| arnot-fd-bdna_X_DC13_DG14 | BB00 | lang-bdna_X_DC13_DG14 | BB00 | aver-bdna_B_DC1_DG2 | NANT |
| arnot-fd-bdna_X_DG14_DC15 | BB00 | lang-bdna_X_DG14_DC15 | BB00 | aver-bdna_B_DG2_DC3 | NANT |
| arnot-fd-bdna_X_DC15_DG16 | BB00 | lang-bdna_X_DC15_DG16 | BB00 | aver-bdna_B_DC3_DG4 | NANT |
| arnot-fd-bdna_X_DG16_DA17 | BB00 | lang-bdna_X_DG16_DA17 | BB00 | aver-bdna_B_DG4_DA5 | NANT |
| arnot-fd-bdna_X_DA17_DA18 | BB00 | lang-bdna_X_DA17_DA18 | BB00 | aver-bdna_B_DA5_DA6 | BB15 |
| arnot-fd-bdna_X_DA18_DT19 | BB00 | lang-bdna_X_DA18_DT19 | BB00 | aver-bdna_B_DA6_DT7 | BB15 |
| arnot-fd-bdna_X_DT19_DT20 | BB00 | lang-bdna_X_DT19_DT20 | BB00 | aver-bdna_B_DT7_DT8 | NANT |
| arnot-fd-bdna_X_DT20_DC21 | BB00 | lang-bdna_X_DT20_DC21 | BB00 | aver-bdna_B_DT8_DC9 | NANT |
| arnot-fd-bdna_X_DC21_DG22 | BB00 | lang-bdna_X_DC21_DG22 | BB00 | aver-bdna_B_DC9_DG10 | NANT |
| arnot-fd-bdna_X_DG22_DC23 | BB00 | lang-bdna_X_DG22_DC23 | BB00 | aver-bdna_B_DG10_DC11 | NANT |
| arnot-fd-bdna_X_DC23_DG24 | BB00 | lang-bdna_X_DC23_DG24 | BB00 | aver-bdna_B_DC11_DG12 | NANT |

### *Journal abbreviations*

| | |
|---|---|
| Proc. Natl. Acad. Sci. USA | PNAS |
| J. Biomol. Struct. Dyn. | JBSD |
| Nucleic Acids Res. | NAR |
| J. Biomol. NMR | JBN |
| J. Am. Chem. Soc. | JACS |
| Biochemistry | BCH |
| Acta Cryst. | Acta |
| J. Mol. Biol. | JMB |
| Chem. Commun. (Camb.) | CHC |
| J. Biol. Chem. | JBC |
| Bioorg. Med. Chem. Lett. | BMCL |

### *Statistics for 1bna + 2bna + 7bna + 9bna*

| Ddnc | count | % |
|---|---|---|
| AA00 | 1 | 1 |
| AB01 | 8 | 9 |
| BA04 | 1 | 1 |
| BA06 | 17 | 19 |
| BA17 | 2 | 2 |
| BB00 | 22 | 25 |
| BB01 | 20 | 23 |
| BB04 | 7 | 8 |
| BB07 | 6 | 7 |
| BB15 | 1 | 1 |
| NANT | 3 | 3 |

**Table S3**. The assignment of the ***NtC*** classes to selected crystal, NMR, and MD structures. c) ***NtC*** classes assigned to dodecamres related to the Dickerson-Drew dodecamer as were deposited to the PDB and after their coordinates were modified by the REDO_PDB protocol. Discussion in the main text was based on ***NtC*** assignment to 34 *D-Dd* structures, Table shows results for selected six structures.

| Step | original confa | original NtC | redo confal | redo NtC |
|---|---|---|---|---|
| 1bna_A_DC_1_DG_2 | 60 | BB04 | 69 | BB04 |
| 1bna_A_DG_2_DC_3 | 91 | BA05 | 84 | BA05 |
| 1bna_A_DC_3_DG_4 | 72 | AB01 | 82 | AB01 |
| 1bna_A_DG_4_DA_5 | 67 | BB04 | 90 | BB00 |
| 1bna_A_DA_5_DA_6 | 75 | BB01 | 94 | BB01 |
| 1bna_A_DA_6_DT_7 | 82 | BA05 | 88 | BA05 |
| 1bna_A_DT_7_DT_8 | 71 | BA05 | 78 | BB01 |
| 1bna_A_DT_8_DC_9 | 69 | BB01 | 81 | BB00 |
| 1bna_A_DC_9_DG_10 | 76 | BB00 | 75 | BB00 |
| 1bna_A_DG_10_DC_11 | 74 | BB07 | 89 | BB07 |
| 1bna_A_DC_11_DG_12 | 73 | BA05 | 69 | BB00 |
| 1bna_B_DC_13_DG_14 | 62 | BB00 | 71 | BB04 |
| 1bna_B_DG_14_DC_15 | 85 | BA05 | 80 | BA05 |
| 1bna_B_DC_15_DG_16 | 73 | AB01 | 80 | AB01 |
| 1bna_B_DG_16_DA_17 | 86 | BB00 | 79 | BB00 |
| 1bna_B_DA_17_DA_18 | 89 | BB00 | 77 | BB00 |
| 1bna_B_DA_18_DT_19 | 87 | BA05 | 87 | BA05 |
| 1bna_B_DT_19_DT_20 | 78 | BB01 | 71 | BB01 |
| 1bna_B_DT_20_DC_21 | 83 | BB01 | 87 | BB01 |
| 1bna_B_DC_21_DG_22 | 70 | BB00 | 71 | BB00 |
| 1bna_B_DG_22_DC_23 | 88 | BA17 | 78 | BA17 |
| 1bna_B_DC_23_DG_24 | 75 | BA05 | 55 | NANT |
| | | | | |
| 1d29_A_DC_1_DG_2 | 66 | BA17 | 78 | BB04 |
| 1d29_A_DG_2_DT_3 | 0 | NANT | 72 | BA01 |
| 1d29_A_DT_3_DG_4 | 0 | NANT | 73 | AB01 |
| 1d29_A_DG_4_DA_5 | 0 | NANT | 80 | BB04 |
| 1d29_A_DA_5_DA_6 | 74 | AB01 | 86 | BB00 |
| 1d29_A_DA_6_DT_7 | 70 | BA01 | 83 | BB01 |
| 1d29_A_DT_7_DT_8 | 0 | NANT | 68 | BB01 |
| 1d29_A_DT_8_DC_9 | 44 | BB01 | 91 | BB00 |
| 1d29_A_DC_9_DA_10 | 54 | BB00 | 72 | BB00 |
| 1d29_A_DA_10_DC_11 | 43 | BB07 | 71 | BB07 |
| 1d29_A_DC_11_DG_12 | 86 | BB00 | 75 | BA01 |
| 1d29_B_DC_13_DG_14 | 0 | NANT | 76 | BB04 |
| 1d29_B_DG_14_DT_15 | 37 | BA05 | 69 | BA01 |
| 1d29_B_DT_15_DG_16 | 0 | NANT | 61 | AB01 |
| 1d29_B_DG_16_DA_17 | 64 | BB04 | 80 | BB04 |
| 1d29_B_DA_17_DA_18 | 71 | BB00 | 93 | BB01 |
| 1d29_B_DA_18_DT_19 | 57 | BB04 | 83 | BB01 |
| 1d29_B_DT_19_DT_20 | 82 | BB01 | 79 | BB00 |
| 1d29_B_DT_20_DC_21 | 70 | BB01 | 88 | BB00 |
| 1d29_B_DC_21_DA_22 | 59 | BB00 | 65 | BB00 |
| 1d29_B_DA_22_DC_23 | 63 | BB07 | 68 | BB07 |
| 1d29_B_DC_23_DG_24 | 62 | BA01 | 71 | NANT |

| | | | | |
|---|---|---|---|---|
| 4i9v_A_DC_1_DG_2 | 89 | BB00 | 90 | BB00 |
| 4i9v_A_DG_2_DC_3 | 39 | BA01 | 43 | BA01 |
| 4i9v_A_DC_3_DG_4 | 73 | AB01 | 76 | AB01 |
| 4i9v_A_DG_4_DA_5 | 95 | BB07 | 93 | BB07 |
| 4i9v_A_DA_5_DA_6 | 79 | BB01 | 77 | BB01 |
| 4i9v_A_DA_6_DT_7 | 91 | BA05 | 91 | BA05 |
| 4i9v_A_DT_7_DT_8 | 82 | BB01 | 81 | BB01 |
| 4i9v_A_DG_10_DC_11 | 83 | BB07 | 85 | BB07 |
| 4i9v_A_DC_11_DG_12 | 67 | BB00 | 65 | BB00 |
| 4i9v_B_DC_13_DG_14 | 95 | BA05 | 94 | BA05 |
| 4i9v_B_DG_14_DC_15 | 68 | BA05 | 68 | BA05 |
| 4i9v_B_DC_15_DG_16 | 92 | AB01 | 92 | AB01 |
| 4i9v_B_DG_16_DA_17 | 77 | BB00 | 76 | BB00 |
| 4i9v_B_DA_17_DA_18 | 92 | BB01 | 90 | BB01 |
| 4i9v_B_DA_18_DT_19 | 94 | BA05 | 92 | BA05 |
| 4i9v_B_DT_19_DT_20 | 68 | BB01 | 69 | BB01 |
| 4i9v_B_DG_22_DC_23 | 97 | BA08 | 97 | BA08 |
| 4i9v_B_DC_23_DG_24 | 85 | AA00 | 87 | AA00 |

| | | | | |
|---|---|---|---|---|
| 5bna_A_DC_1_DG_2 | 62 | BB00 | 0 | NANT |
| 5bna_A_DG_2_DC_3 | 76 | BA05 | 65 | BA05 |
| 5bna_A_DC_3_DG_4 | 68 | AB01 | 78 | AB01 |
| 5bna_A_DG_4_DA_5 | 0 | NANT | 71 | BB00 |
| 5bna_A_DA_5_DA_6 | 64 | BB00 | 93 | BB00 |
| 5bna_A_DA_6_DT_7 | 52 | BB01 | 62 | BA05 |
| 5bna_A_DT_7_DT_8 | 78 | BA05 | 80 | BA05 |
| 5bna_A_DT_8_DC_9 | 70 | BA05 | 82 | BB01 |
| 5bna_A_DC_9_DG_10 | 71 | AB01 | 47 | BB00 |
| 5bna_A_DG_10_DC_11 | 80 | BB07 | 73 | BB07 |
| 5bna_A_DC_11_DG_12 | 53 | BB00 | 0 | NANT |
| 5bna_B_DC_13_DG_14 | 65 | BB07 | 63 | BB04 |
| 5bna_B_DG_14_DC_15 | 78 | BA05 | 68 | BA05 |
| 5bna_B_DC_15_DG_16 | 65 | AB01 | 0 | NANT |
| 5bna_B_DG_16_DA_17 | 58 | BB00 | 56 | BB00 |
| 5bna_B_DA_17_DA_18 | 62 | BB01 | 64 | BB01 |
| 5bna_B_DA_18_DT_19 | 0 | NANT | 68 | NANT |
| 5bna_B_DT_19_DT_20 | 0 | NANT | 65 | NANT |
| 5bna_B_DT_20_DC_21 | 65 | BB01 | 73 | BB01 |
| 5bna_B_DC_21_DG_22 | 79 | BB00 | 53 | BB00 |
| 5bna_B_DG_22_DC_23 | 45 | BA08 | 60 | BA17 |
| 5bna_B_DC_23_DG_24 | 56 | AA00 | 68 | AA03 |

*DNA models derived from fiber diffraction.*

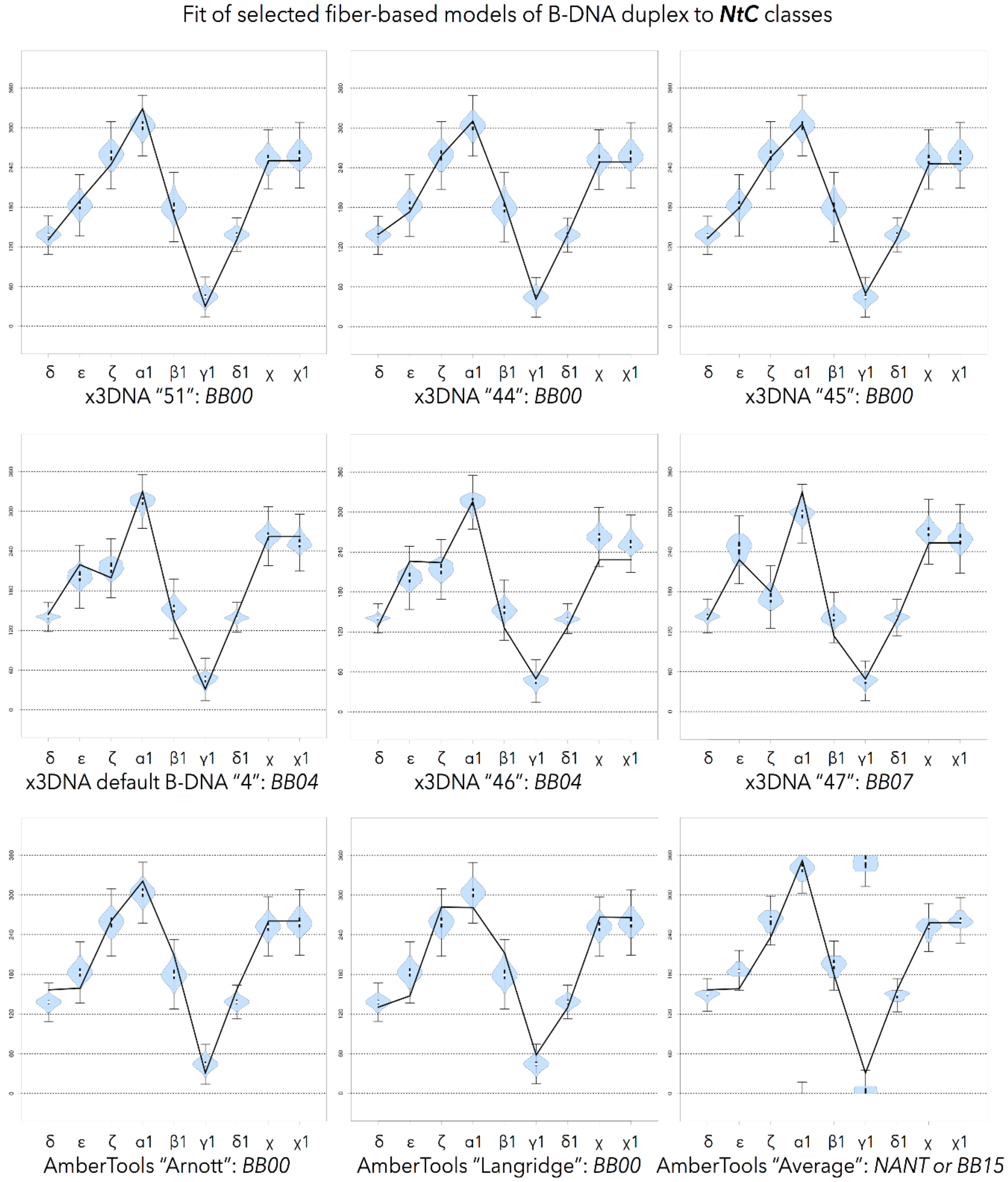


**Figure S4**. Assignment of ***NtC*** classes for B-DNA duplex models based on the fiber data. Shown are assignments of six models from x3DNA (Lu & Olson, 2008) and three models from Nucleic Acid Builder of AmberTools14 (Salomon-Ferrer *et al.*, 2013).

**Table S4**. The assignment of ***NtC*** classes to the fiber model structures, which are available in x3DNA.

| x3dna description of the fiber model | | | | Assigned dinucleotide conformers | | | | | | | | Comments |
|---|---|---|---|---|---|---|---|---|---|---|---|---|
| id# | Twist [°] | Rise [Å] | Structure description | Ddnc | % | Ddnc | % | Ddnc | % | Ddnc | % | |
| 1 | 33 | 2.55 | A-DNA (calf thymus; generic sequence: A, C, G and T) | AA00 | 100 | | | | | | | **default A form fiber in x3dna** |
| 2 | 66 | 5.10 | A-DNA poly d(ABr5U) : poly d(ABr5U) | AA00 | 100 | | | | | | | |
| 3 | 0 | 28.03 | A-DNA (calf thymus) poly d(A1T2C3G4G5A6A7T8G9G10T11) poly d(A1C2C3A4T5T6C7C8G9A10T11) | AA00 | 100 | | | | | | | |
| 4 | 36 | 3.38 | B-DNA (calf thymus; generic sequence: A, C, G and T) | BB04 | 100 | | | | | | | **default B form fiber in x3dna** |
| 5 | 72 | 6.72 | B-DNA poly d(CG) : poly d(CG) | BB07 | 50 | BB04 | 50 | | | | | |
| 6 | 180 | 16.86 | B-DNA (calf thymus) poly d(C1C2C3C4C5) : poly d(G6G7G8G9G10) | BB00 | 39 | BB07 | 33 | BB04 | 28 | | | chain A (BB00+BB07), chain B (BB00+BB07+BB04) |
| 7 | 39 | 3.31 | C-DNA (calf thymus; generic sequence: A, C, G and T) | BB00 | 100 | | | | | | | |
| 8 | 40 | 3.31 | C-DNA poly d(GGT) : poly d(ACC) | BB07 | 100 | | | | | | | |
| 9 | 120 | 9.94 | C-DNA poly d(G1G2T3) : poly d(A4C5C6) | BB04 | 30 | BB07 | 30 | BB00 | 20 | NANT | 20 | chain A (BB04, BB00), chain B (BB07, NANT), NANT similar to BB07 (zeta too high) |
| 10 | 80 | 6.47 | C-DNA poly d(AG) : poly d(CT) | BB00 | 67 | NANT | 33 | | | | | NANT similar to BB04 (gamma too high) |
| 11 | 80 | 6.47 | C-DNA poly d(A1G2) : poly d(C3T4) | BB04 | 33 | BB00 | 33 | BB07 | 17 | NANT | 17 | NANT similar to BB16 (zeta too low, gamma and chi too high) |
| 12 | 45 | 3.01 | D-DNA poly d(AAT) : poly d(ATT) | BB04 | 100 | | | | | | | |
| 13 | 90 | 6.13 | D-DNA poly d(CI) : poly d(CI) | BB04 | 100 | | | | | | | |
| 14 | -90 | 18.50 | D-DNA poly d(A1T2A3T4A5T6) : poly d(A1T2A3T4A5T6) | BB00 | 55 | NANT | 36 | BB04 | 9 | | | NANT similar to BB07 (alpha too low, zeta and gamma too high) |
| 15 | -60 | 7.25 | Z-DNA poly d(GC) : poly d(GC) | ZZS1 | 67 | ZZ1S | 33 | | | | | default Z form fiber in x3dna |
| 16 | -51 | 7.57 | Z-DNA poly d(As4T) : poly d(As4T) | ZZS1 | 67 | ZZ1S | 33 | | | | | |
| 17 | 0 | 10.20 | L-DNA (calf thymus) poly d(GC) : poly d(GC) | NANT | 100 | | | | | | | weird torsions (76 171 124 298 227 221 147 26 167 and 147 276 98 82 198 180 76 167 26) |
| 18 | 36 | 3.23 | B'-DNA alpha poly d(A) : poly d(T) (H-DNA) | BB07 | 50 | BB04 | 50 | | | | | chain A (BB07), chain B (BB04) |
| 19 | 36 | 3.23 | B'-DNA beta2 poly d(A) : poly d(T) (H-DNA beta) | AA00 | 50 | BB00 | 50 | | | | | chain A (AA00), chain B (BB00) |
| 20 | 33 | 2.81 | A-RNA poly (A) : poly (U) | AA00 | 100 | | | | | | | |
| 21 | 30 | 3.00 | A'-RNA poly (I) : poly (C) | AA00 | 100 | | | | | | | |
| 22 | 33 | 2.56 | Hybrid poly (A) : poly d(T) | AA00 | 100 | | | | | | | |
| 23 | 32 | 2.78 | Hybrid poly d(G) : poly (C) | AA00 | 100 | | | | | | | |
| 24 | 36 | 3.13 | Hybrid poly d(I) : poly (C) | AA00 | 100 | | | | | | | |
| 25 | 33 | 3.06 | Hybrid poly d(A) : poly (U) | BB00 | 50 | AA00 | 50 | | | | | chain A (BB00), chain B (AA00) |
| 26 | 36 | 3.01 | 10-fold poly (X) : poly (X) | - | | | | | | | | non-standard base, not assigned |
| 27 | 33 | 2.52 | 11-fold poly (X) : poly (X) | - | | | | | | | | non-standard base, not assigned |
| 28 | 33 | 2.60 | Poly (s2U) : poly (s2U) (symmetric base-pair) | AA00 | 100 | | | | | | | |
| 29 | 33 | 2.60 | Poly (s2U) : poly (s2U) (asymmetric base-pair) | AA00 | 100 | | | | | | | |
| 30 | 33 | 3.16 | Poly d(C) : poly d(I) : poly d(C) | AA00 | 100 | | | | | | | |
| 31 | 30 | 3.26 | Poly d(T) : poly d(A) : poly d(T) | AA00 | 100 | | | | | | | |
| 32 | 33 | 3.04 | Poly (U) : poly (A) : poly(U) (11-fold) | AA00 | 100 | | | | | | | |
| 33 | 30 | 3.04 | Poly (U) : poly (A) : poly(U) (12-fold) | AA00 | 67 | AA03 | 33 | | | | | |
| 34 | 30 | 3.29 | Poly (I) : poly (A) : poly(I) | AA00 | 100 | | | | | | | |
| 35 | 31 | 3.41 | Poly (I) : poly (I) : poly(I) : poly(I) | AA04 | 100 | | | | | | | |
| 36 | 60 | 3.16 | Poly (C) or poly (mC) or poly (eC) | AA00 | 100 | | | | | | | |
| 37 | 36 | 3.20 | B'-DNA beta2 Poly d(A) : poly d(U) | BB04 | 50 | BB00 | 50 | | | | | chain A (BB04), chain B (BB00) |
| 38 | 36 | 3.24 | B'-DNA beta1 Poly d(A) : poly d(T) | BB07 | 50 | BB04 | 50 | | | | | chain A (BB07), chain B (BB04) |
| 39 | 72 | 6.48 | B'-DNA beta2 Poly d(AI) : poly d(CT) | BB00 | 50 | BB01 | 50 | | | | | 0101 within chain |
| 40 | 72 | 6.46 | B'-DNA beta1 Poly d(AI) : poly d(CT) | BB04 | 50 | BB00 | 50 | | | | | 4040 within chain |
| 41 | 144 | 13.54 | B'-DNA Poly d(AATT) : poly d(AATT) | BB00 | 51 | BB04 | 26 | BB07 | 23 | | | ~ 0470 within chain |
| 42 | 33 | 3.04 | Poly(U) : poly d(A) : poly(U) [cf. #32] | AA00 | 100 | | | | | | | |
| 43 | 36 | 3.20 | Beta Poly d(A) : Poly d(U) [cf. #37] | BB04 | 50 | BB00 | 50 | | | | | chain A (BB04), chain B (BB00) |
| 44 | 36 | 3.23 | Poly d(A) : poly d(T) (Ca salt) | BB00 | 100 | | | | | | | |
| 45 | 36 | 3.23 | Poly d(A) : poly d(T) (Na salt) | BB00 | 100 | | | | | | | |
| 46 | 36 | 3.38 | B-DNA (BI-type nucleotides; generic sequence: A, C, G and T) | BB04 | 100 | | | | | | | |
| 47 | 40 | 3.32 | C-DNA (BII-type nucleotides; generic sequence: A, C, G and T) | BB07 | 100 | | | | | | | default BII form fiber in x3dna |
| 48 | 88 | 6.02 | D(A)-DNA ploy d(AT) : ploy d(AT) (right-handed) | AA00 | 100 | | | | | | | |
| 49 | 60 | 7.20 | S-DNA ploy d(CG) : poly d(CG) (C_BG_A, right-handed) | BA01 | 50 | AB03 | 50 | | | | | |
| 50 | 60 | 7.20 | S-DNA ploy d(GC) : poly d(GC) (C_AG_B, right-handed) | BA01 | 50 | AB03 | 50 | | | | | |
| 51 | 32 | 3.22 | B*-DNA poly d(A) : poly d(T) | BB00 | 100 | | | | | | | |
| 52 | 90 | 6.06 | D(B)-DNA poly d(AT) : poly d(AT) [cf. #48] | BB04 | 100 | | | | | | | |
| 53 | -39 | 3.29 | C-DNA (generic sequence: A, C, G and T) (depreciated) | NANT | 100 | | | | | | | strange torsions (147 290.3 176.2 58.9 220.2 157.6 147 177.1 177.1) |
| 54 | 33 | 2.56 | A-DNA (generic sequence: A, C, G and T) [cf. #1] | AA00 | 100 | | | | | | | |
| 55 | 36 | 3.39 | B-DNA (generic sequence: A, C, G and T) [cf. #4] | BB04 | 100 | | | | | | | |

**References**

Models #1 to #41 are based on Struther Arnott: ``Polynucleotide secondary structures: an historical perspective'', pp. 1-38 in ``Oxford Handbook of Nucleic Acid Structure'' edited by Stephen Neidle (Oxford Press, 1999).
Models #42 and #43 are from Chandrasekaran & Arnott: "The Structures of DNA and RNA Helices in Oriented Fibers", pp 31-170 in "Landolt-Bornstein Numerical Data and Functional edited by W. Saenger (Springer-Verlag, 1990).
Models #44-#45 based on Alexeev et al., ``The structure of poly(dA) . poly(dT) as revealed by an X-ray fiber diffraction''. J. Biomol. Str. Dyn, 4, pp. 989-1011, 1987.
Models #46-#47 based on van Dam & Levitt, ``BII nucleotides in the B and C forms of natural-sequence polymeric DNA: a new model for the C form of DNA''. J. Mol. Biol., 304, pp. 541-561, 2000.
Models #48-#55 based on Premilat & Albiser, ``A new D-DNA form of poly(dA-dT).poly(dA-dT): an A-DNA type structure with reversed Hoogsteen Pairing''. Eur. Biophys. J., 30, pp. 404-410, 2001 (and several other publications).

### *References for the supplement*

Adhireksan, Z., Davey, G. E., Campomanes, P., Groessl, M., Clavel, C. M., Yu, H., Nazarov, A. A., Yeo, C. H., Ang, W. H., Droge, P., Rothlisberger, U., Dyson, P. J. & Davey, C. A. (2014). *Nat. Commun.* **5**, 3462.

Ahlrichs, R., Bar, M., Haser, M., Horn, H. & Kolmel, C. (1989). *Chem. Phys. Lett.* **162**, 165-169.

Aymami, J., Nunn, C. M. & Neidle, S. (1999). *Nucleic Acids Res.* **27**, 2691-2698.

Barabas, O., Ronning, D. R., Guynet, C., Hickman, A. B., Ton-Hoang, B., Chandler, M. & Dyda, F. (2008). *Cell* **132**, 208-220.

Boer, R., Russi, S., Guasch, A., Lucas, M., Blanco, A. G., Perez-Luque, R., Coll, M. & de la Cruz, F. (2006). *J. Mol. Biol.* **358**, 857-869.

Collie, G. W., Promontorio, R., Hampel, S. M., Micco, M., Neidle, S. & Parkinson, G. N. (2012). *J. Am. Chem. Soc.* **134**, 2723-2731.

Davis, I. W., Leaver-Fay, A., Chen, V. B., Block, J. N., Kapral, G. J., Wang, X., Murray, L. W., Arendall, W. B., 3rd, Snoeyink, J., Richardson, J. S. & Richardson, D. C. (2007). *Nucleic Acids Res.* **35**, W375-383.

Frouws, T. D., Duda, S. C. & Richmond, T. J. (2016). *Proc. Natl. Acad. Sci. USA* **113**, 1214-1219.

Gao, Y. G., Robinson, H., Wijsman, E. R., van der Marel, G. A., van Boom, J. H. & Wang, A. H.-J. (1997). *J. Am. Chem. Soc.* **119**, 1496-1497.

Glas, A. F., Maul, M. J., Cryle, M., Barends, T. R., Schneider, S., Kaya, E., Schlichting, I. & Carell, T. (2009). *Proc. Natl. Acad. Sci. USA* **106**, 11540-11545.

Gleghorn, M. L., Davydova, E. K., Rothman-Denes, L. B. & Murakami, K. S. (2008). *Mol. Cell* **32**, 707-717.

Goedecke, K., Pignot, M., Goody, R. S., Scheidig, A. J. & Weinhold, E. (2001). *Nat. Struct. Biol.* **8**, 121-125.

Haider, S., Parkinson, G. N. & Neidle, S. (2002). *J.Mol.Biol.* **320**, 189-200.

Chen, Y. & Rice, P. A. (2003). *J. Biol. Chem.* **278**, 24800-24807.

Chevalier, B. S., Kortemme, T., Chadsey, M. S., Baker, D., Monnat, R. J. & Stoddard, B. L. (2002). *Mol. Cell* **10**, 895-905.

Johnson, S. J. & Beese, L. S. (2004). *Cell* **116**, 803-816.

Jurecka, P., Cerny, J., Hobza, P. & Salahub, D. R. (2007). *J. Comput. Chem.* **28**, 555-569.

Kondo, J., Yamada, T., Hirose, C., Okamoto, I., Tanaka, Y. & Ono, A. (2014). *Angew. Chem. Int. Ed. Engl.* **53**, 2385-2388.

Larkin, M. A., Blackshields, G., Brown, N. P., Chenna, R., McGettigan, P. A., McWilliam, H., Valentin, F., Wallace, I. M., Wilm, A., Lopez, R., Thompson, J. D., Gibson, T. J. & Higgins, D. G. (2007). *Bioinformatics* **23**, 2947-2948.

Lu, X. J. & Olson, W. K. (2008). *Nat. Protoc.* **3**, 1213-1227.

McAuley-Hecht, K. E., Leonard, G. A., Gibson, N. J., Thomson, J. B., Watson, W. P., Hunter, W. N. & Brown, T. (1994). *Biochemistry* **33**, 10266-10270.

Mondragon, A. & Harrison, S. C. (1991). *J. Mol. Biol.* **219**, 321-334.

Nicoludis, J. M., Miller, S. T., Jeffrey, P. D., Barrett, S. P., Rablen, P. R., Lawton, T. J. & Yatsunyk, L. A. (2012). *J. Am. Chem. Soc.* **134**, 20446-20456.

Parkinson, G. N., Lee, M. P. & Neidle, S. (2002). *Nature* **417**, 876-880.

Patikoglou, G. A., Kim, J. L., Sun, L., Yang, S. H., Kodadek, T. & Burley, S. K. (1999). *Genes Dev.* **13**, 3217-3230.

Salomon-Ferrer, R., Case, D. A. & Walker, R. C. (2013). *Wiley Interdisciplinary Reviews: Computational Molecular Science* **3**, 198-210.

Schafer, A., Huber, C. & Ahlrichs, R. (1994). *J. Chem. Phys.* **100**, 5829-5835.

Schellenberg, M. J., Appel, C. D., Adhikari, S., Robertson, P. D., Ramsden, D. A. & Williams, R. S. (2012). *Nat. Struct. Mol. Biol.* **19**, 1363-1371.
Sriram, M., van der Marel, G. A., Roelen, H. L. P. F., van Boom, J. H. & Wang, A. H.-J. (1992). *Biochemistry* **31**, 11823-11834.
Svozil, D., Kalina, J., Omelka, M. & Schneider, B. (2008). *Nucleic Acids Res.* **36**, 3690-3706.
Swan, M. K., Johnson, R. E., Prakash, L., Prakash, S. & Aggarwal, A. K. (2009). *J. Mol. Biol.* **390**, 699-709.
Tao, J., Perdew, J. P., Staroverov, V. N. & Scuseria, G. E. (2003). *Phys. Rev. Lett.* **91**, 146401.
Temperini, C., Messori, L., Orioli, P., Di Bugno, C., Animati, F. & Ughetto, G. (2003). *Nucleic Acids Res.* **31**, 1464-1469.
Thorpe, J. H., Gale, B. C., Teixeira, S. C. & Cardin, C. J. (2003). *J. Mol. Biol.* **327**, 97-109.
Vasudevan, D., Chua, E. Y. & Davey, C. A. (2010). *J. Mol. Biol* **403**, 1-10.
Wang, M., Xia, S., Blaha, G., Steitz, T. A., Konigsberg, W. H. & Wang, J. (2011). *Biochemistry* **50**, 581-590.
Williams, R. S., Moncalian, G., Williams, J. S., Yamada, Y., Limbo, O., Shin, D. S., Groocock, L. M., Cahill, D., Hitomi, C., Guenther, G., Moiani, D., Carney, J. P., Russell, P. & Tainer, J. A. (2008). *Cell* **135**, 97-109.
Wilson, R. C., Jackson, M. A. & Pata, J. D. (2013). *Structure* **21**, 20-31.
Xia, S., Wang, J. & Konigsberg, W. H. (2013). *J. Am. Chem. Soc.* **135**, 193-202.
